# Supplementary material for: Competition and evolutionary selection among core regulatory motifs in gene expression control
Source: Nat Commun. 2023 Dec 13;14:8266. doi: 10.1038/s41467-023-43327-7 (PMC10719253; doi:10.1038/s41467-023-43327-7)
Supplement: Supplementary file 1 — Supplementary Information [file 41467_2023_43327_MOESM1_ESM.pdf]

Competition and evolutionary selection among core regulatory motifs in gene expression control

Supplementary Information

Andras Gyorgy[1]*

[1]Division of Engineering, New York University Abu Dhabi, Abu Dhabi, UAE
*To whom correspondence should be addressed; E-mail: andras.gyorgy@nyu.edu.

This Supplementary Information includes additional simulation data and further details on (i) the mechanistic model underpinning the core regulatory motifs; (ii) fitness cost in large populations; (iii) fitness cost in small populations; (iv) how autoregulation can result in unwanted selection pressure; (v) how delay can cause non-autoregulated motifs to outperform autoregulation; (vi) how feedback cost can cause non-autoregulated motifs to outperform autoregulation; (vii) how reduced bioenergetic cost may contribute to the prevalence of autoregulation in *B. subtilis*, *C. glutamicum*, and *E. coli*; and (viii) how autoregulation can impact genetic stability in synthetic biology applications.

Contents

1 Mathematical model **2**

2 Fitness cost in large populations **8**

3 Fitness cost in small populations **22**

4 Autoregulation can result in unwanted selection pressure **27**

5 Delay can cause non-autoregulated motifs to outperform autoregulation **30**

6 Feedback cost can cause non-autoregulated motifs to outperform autoregulation **37**

7 Reduced bioenergetic cost may contribute to the prevalence of autoregulation . **40**

8 Autoregulation can impact genetic stability in synthetic biology applications . . **46**

1 Mathematical model

Here, we present the mechanistic models underpinning the control schemes featured in Fig. 1. We demonstrate that while the regulator R is continuously expressed throughout the entire period for non-autoregulated motifs, its expression is only turned on during the induced phase for autoregulation. Following this, we detail how fitness cost can be mapped to reduced growth rate, and illustrate that autoregulation can offer superior performance by eliminating the gratuitous expression of the regulator R. It is important to note that the results in subsequent sections and in the main text do not depend on the exact details of the models presented here. Instead, they are underpinned by the time-varying selection pressures featured in Fig. 2c. As a result, the particular modeling choices can be flexibly modified without impacting the findings to capture more complex phenomena, for instance, the multimerization of the regulator or the impact of shared cellular resources and the resulting metabolic burden.

1.1 Positive control

The dynamics of R and P when the former is not autoregulated are given by

$$\frac{\mathrm{d}R}{\mathrm{d}t} = \alpha_R - \delta_R R, \qquad \frac{\mathrm{d}P}{\mathrm{d}t} = \alpha_P \frac{R + \epsilon K(I)}{R + K(I)} - \delta_P P,$$

where α_R and α_P are production rate constants, $\epsilon \ll 1$ captures promoter leakiness, δ_R and δ_P are degradation rate constants, and $K(I)$ denotes the dissociation constant of the activator binding to its cognate promoter. Let K_{ni} and K_i denote the value of $K(I)$ in the absence and presence of the inducer I during the non-induced and induced phases, respectively, such that $K_{ni} \gg K_i$ (i.e., binding is stronger in the induced phase). Similarly, the dynamics are given by

$$\frac{\mathrm{d}R}{\mathrm{d}t} = \bar{\alpha}_R \frac{R + \epsilon K(I)}{R + K(I)} - \delta_R R, \qquad \frac{\mathrm{d}P}{\mathrm{d}t} = \alpha_P \frac{R + \epsilon K(I)}{R + K(I)} - \delta_P P$$

when R is self-activated, where $\bar{\alpha}_R$ is the production rate constant.

Introduce $\tau = t\delta_P$, $r = R\delta_R/\alpha_R$, $p = P\delta_P/\alpha_P$, $k(I) = K(I)\delta_R/\alpha_R$, $k_i = K_i\delta_R/\alpha_R$, $k_{ni} = K_{ni}\delta_R/\alpha_R$, and $\bar{\alpha} = \bar{\alpha}_R/\alpha_R$. The de-dimensionalized dynamics for non-autoregulated activation thus become

$$\frac{\mathrm{d}r}{\mathrm{d}\tau} = \gamma\left(1 - r\right), \qquad \frac{\mathrm{d}p}{\mathrm{d}\tau} = \frac{r + \epsilon k(I)}{r + k(I)} - p, \tag{1}$$

while for self-activation we obtain

$$\frac{\mathrm{d}r}{\mathrm{d}\tau} = \gamma\left(\bar{\alpha}\frac{r + \epsilon k(I)}{r + k(I)} - r\right), \qquad \frac{\mathrm{d}p}{\mathrm{d}\tau} = \frac{r + \epsilon k(I)}{r + k(I)} - p, \tag{2}$$

where $\gamma = \delta_R/\delta_P$ captures the timescale difference between the dynamics of the regulator and the product. At the steady state we have $r = 1$ in the absence of autoregulation, whereas in its presence we obtain

$$r = \frac{(\bar{\alpha} - k(I)) \pm \sqrt{(\bar{\alpha} - k(I))^2 + 4\epsilon\bar{\alpha}k(I)}}{2},$$

hence there is a unique positive equilibrium. During the non-induced phase we have $r \to \epsilon\bar{\alpha}$ as $k_{ni} \to \infty$, and during the induced phase we obtain $r \to \bar{\alpha}$ as $k_i \to 0$. Therefore, we can approximate the expression of the regulator R being off/on during the non-induced/induced phases when $\epsilon \ll 1$ (yielding negligible basal expression in the non-induced phase, Fig. 1b).

To ensure that r and p take on the same values for non-autoregulated and autoregulated positive control during the induced phase, note first that we have $p = [r + \epsilon k(I)]/[r + k(I)]$ in both cases at the equilibrium. Therefore, it is sufficient to ensure that r takes on the same value in the absence and presence of autoregulation to guarantee identical steady state behavior of p. This requirement yields the constraint

$$0 = \bar{\alpha}\frac{1 + \epsilon k(I)}{1 + k(I)} - 1,$$

hence by choosing $\bar{\alpha} = (1 + k_i)/(1 + \epsilon k_i)$ we can ensure that the values of both r and p (thus the concentrations of R and P) are identical in the presence of the inducer (i.e., during the induced phase) for non-autoregulated and autoregulated positive control of the regulator.

1.2 Negative control

The dynamics of R and P when the former is not autoregulated are given by

$$\frac{\mathrm{d}R}{\mathrm{d}t} = \alpha_R - \delta_R R, \qquad \frac{\mathrm{d}P}{\mathrm{d}t} = \alpha_P \frac{\epsilon R + K(I)}{R + K(I)} - \delta_P P,$$

where $K(I)$ now denotes the dissociation constant of the repressor binding to its cognate promoter (all other parameters are defined as before). Let K_{ni} and K_i denote the value of $K(I)$ in the absence and presence of the inducer I during the non-induced and induced phases, respectively, such that $K_{ni} \ll K_i$ (i.e., binding is weaker in the induced phase). Similarly, when R is self-repressed, the dynamics are given by

$$\frac{\mathrm{d}R}{\mathrm{d}t} = \bar{\alpha} \frac{\epsilon R + K(I)}{R + K(I)} - \delta_R R, \qquad \frac{\mathrm{d}P}{\mathrm{d}t} = \alpha_P \frac{\epsilon R + K(I)}{R + K(I)} - \delta_P P,$$

where $\bar{\alpha}_R$ is the production rate constant. With the same de-dimensionalization steps as for positive control, for non-autoregulated repression we obtain the dynamics

$$\frac{\mathrm{d}r}{\mathrm{d}\tau} = \gamma\left(1 - r\right), \qquad \frac{\mathrm{d}p}{\mathrm{d}\tau} = \frac{\epsilon r + k(I)}{r + k(I)} - p, \tag{3}$$

whereas for self-repression we have

$$\frac{\mathrm{d}r}{\mathrm{d}\tau} = \gamma\left(\bar{\alpha}\frac{\epsilon r + k(I)}{r + k(I)} - r\right), \qquad \frac{\mathrm{d}p}{\mathrm{d}\tau} = \frac{\epsilon r + k(I)}{r + k(I)} - p. \tag{4}$$

Thus, at the steady state we obtain $r = 1$ in the absence of autoregulation, whereas in its presence $r = [(\epsilon\bar{\alpha} - k(I)) \pm \sqrt{(\epsilon\bar{\alpha} - k(I))^2 + 4\bar{\alpha}k(I)}]/2$, with a unique positive root, such that $r \to \epsilon\bar{\alpha}$ as $k_{ni} \to 0$ during the non-induced phase and $r \to \bar{\alpha}$ as $k_i \to \infty$ during the induced phase. Therefore, we can approximate the expression of the regulator R being off/on during the non-induced/induced phases when $\epsilon \ll 1$ (yielding negligible basal expression in the non-induced phase, Fig. 1b). Finally, to ensure that both r and p (thus the concentration of R and P) match in the presence of the inducer (i.e., during the induced phase) for non-autoregulated and autoregulated negative control of the regulator, it is sufficient to choose $\bar{\alpha}_i = (1 + k_i)/(\epsilon + k_i)$.

1.3 Fitness cost and growth rate

To relate fitness cost to the resulting growth rate reduction, consider two competing variants. Normalize time so that the growth rate of variant #1 is precisely 1, and let $g < 1$ denote the growth rate of variant #2, reduced due to its fitness cost s when compared to variant #1. Members of variant #2 produce fewer offsprings than those of variant #1, leading to a reduced fraction x of the former in the population. Considering the standard Wright-Fisher model of population genetics (*1*), this can be modeled as $x \to x - sx(1-x)/(1-sx)$. Alternatively, during an average generation of variant #1 ($\Delta t = 1$), the fraction x of the population that represents variant #2 changes as $x \to xe^g/[xe^g + (1-x)xe]$. Taken together, we obtain $g = G(s)$ with

$$G(s) = \begin{cases} 1 + \ln(1-s) & \text{, if } s < 1 - 1/e, \\ 0 & \text{, otherwise,} \end{cases} \tag{5}$$

connecting fitness cost and the resulting reduction in growth rate ($g = 0$ corresponds to growth arrest in the presence of sufficiently high fitness cost). Importantly, (5) is only used to illustrate competition between non-autoregulated and autoregulated motifs in Supplementary Fig. 1–Supplementary Fig. 3, other results presented in subsequent sections and in the main text are independent of it.

1.4 Autoregulation can dominate non-autoregulated control

In Supplementary Fig. 1–Supplementary Fig. 2 we present detailed simulation data considering $s_p = \lambda_p|p_{\text{ref}} - p|$ and $s_r = \lambda_r r$, where $\lambda_p, \lambda_r > 0$ and p_{ref} is the reference signal for the product: $p_{\text{ref}} = 1$ during the induced phase, and $p_{\text{ref}} = 0$ during the non-induced phase. In the absence of mutations, growth rate is captured via $g = G(s)$ from (5) with $s = s_p + s_r$, together with the growth law $\dot{N} = Ng$, where N is the population size. These results confirm that (i) the control schemes work as expected, and (ii) autoregulated motifs can outperform their non-autoregulated counterparts by eliminating the gratuitous expression of the regulator.

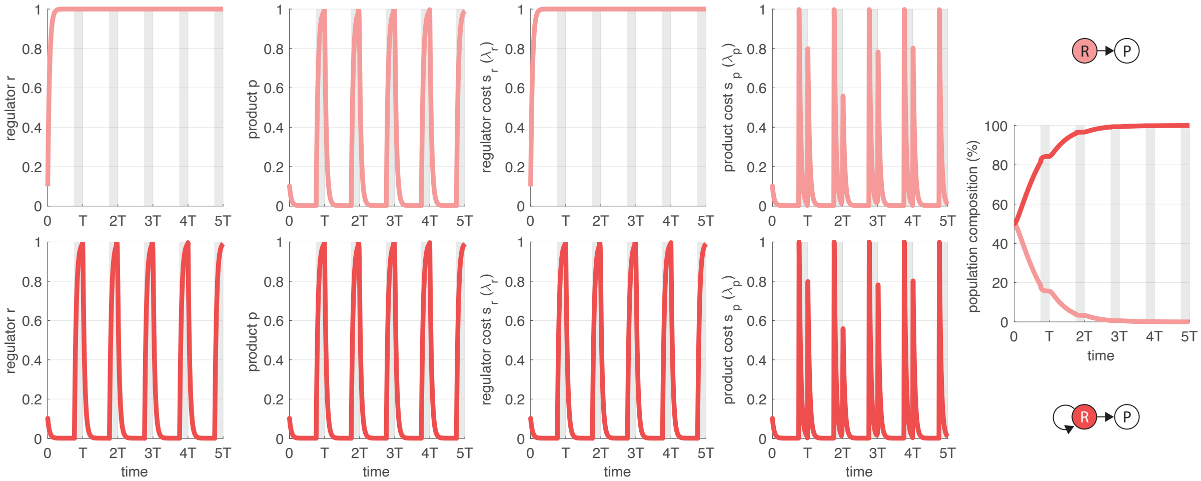

Supplementary Fig. 1. **Autoregulation eliminates the gratuitous expression of the regulator, thus reducing the associated fitness cost (positive control).** Light and dark red corresponds to non-autoregulated and autoregulated activation, respectively. Induced and non-induced phases alternate with a period of $T = 20\gamma$, the former takes up 25% of each period (gray shaded regions). Simulation parameters are: $k_i = 10^{-3}$, $k_{ni} = 10^3$, $\epsilon = 10^{-3}$, $\gamma = 1$, $\lambda_p = 0.3$, $\lambda_r = 0.1$.

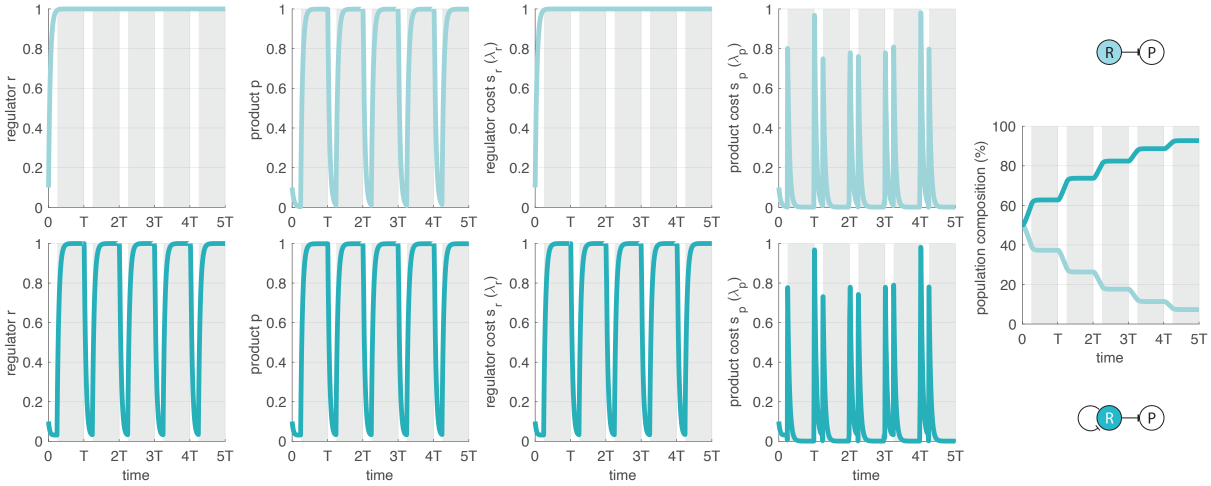

Supplementary Fig. 2. **Autoregulation eliminates the gratuitous expression of the regulator, thus reducing the associated fitness cost (negative control).** Light and dark blue corresponds to non-autoregulated and autoregulated repression, respectively. Induced and non-induced phases alternate with a period of $T = 20\gamma$, the former takes up 75% of each period (gray shaded regions). Simulation parameters are: $k_i = 10^{-3}$, $k_{ni} = 10^3$, $\epsilon = 10^{-3}$, $\gamma = 1$, $\lambda_p = 0.3$, $\lambda_r = 0.1$.

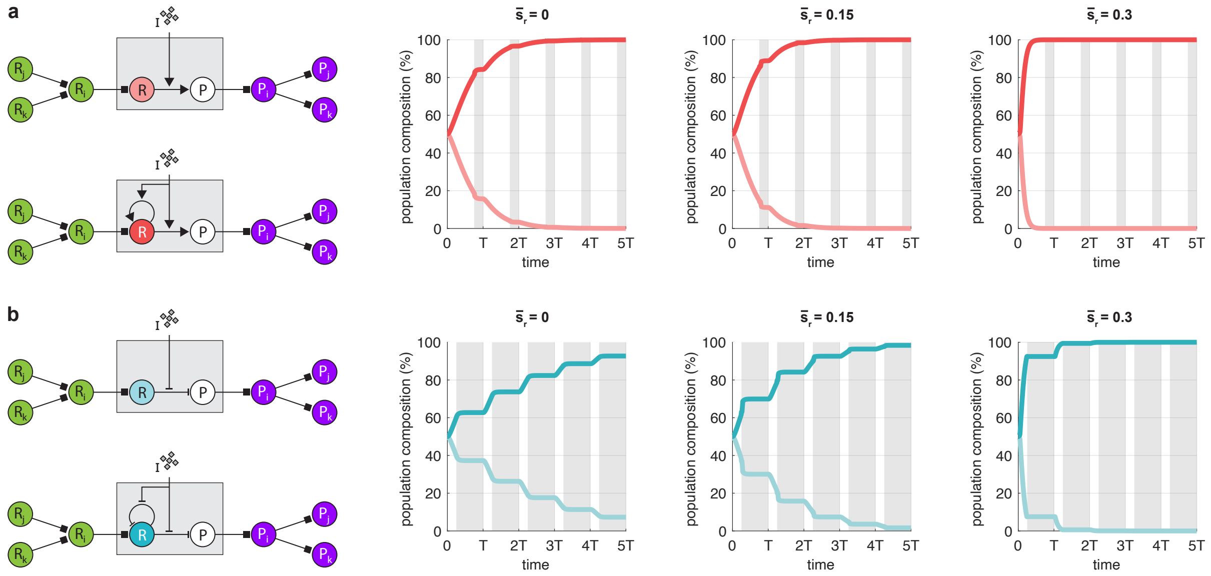

Supplementary Fig. 3. **The core regulatory motifs may be embedded in complex pathways.** Gray squares denote the inducer I. Light and dark red/blue corresponds to non-autoregulated and autoregulated activator/repressor, respectively. Green/purple indicates elements of the pathway upstream/downstream of the motif. Arrows with square arrowheads represent either activation or repression. Induced (gray shaded regions) and non-induced phases alternate with a period of $T = 20\gamma$. Simulation parameters are: $k_i = 10^{-3}$, $k_{ni} = 10^3$, $\epsilon = 10^{-3}$, $\gamma = 1$, $\lambda_p = 0.3$, $\lambda_r = 0.1$. **a** Demand is $D = 0.25$. **b** Demand is $D = 0.75$.

In Supplementary Fig. 3 we illustrate what happens when the motifs from Fig. 1 are embedded in a complex pathway. In particular, R may be regulated by an upstream process comprising R_i, R_j, and R_k, with the bioenergetic cost $\bar{s}_r(t)$ corresponding to their expression. As a result, the total fitness cost becomes $s = s_p + s_r + \bar{s}_r$, where the P-cost s_p may depend not only on P regulated directly by R, but also on other species downstream of it represented by P_i, P_j, and P_k. As identical upstream and downstream pathways yield identical $\bar{s}_r$ and s_p for all motifs considering the same concentrations of the regulator and the product, the relative fitness difference among them remains unchanged once they are embedded within their context. Therefore, autoregulated schemes still emerge as dominant (Supplementary Fig. 3), the additional fitness cost $\bar{s}_r$ impacts only the rate at which this dominance is established according to $G(s)$ in (5).

2 Fitness cost in large populations

In populations of considerable size, the impact of stochastic fluctuations is negligible. Thus, the effects of mutation and selection can be characterized by relying on a deterministic approach (*1*), considering the dynamics

$$\begin{aligned}\dot{N}_b =& \nu_+ N_{nb} - \nu_- N_b - s_b N_b,\\ \dot{N}_{nb} =& \nu_- N_b - \nu_+ N_{nb} - s_{nb} N_{nb},\end{aligned} \tag{6}$$

where N_b and N_{nb} denote the number of binders and non-binders (Fig. 2b), together with the corresponding time-varying fitness costs s_b and s_{nb}, and with the gain-of-function and loss-of-function mutation rates ν_+ and ν_-, respectively. Introducing $x = N_{nb}/(N_n + N_{nb})$ to denote the fraction of non-binders in the population, from (6) we obtain

$$\dot{x} = \nu_- - x\,(\nu + s) + x^2 s \tag{7}$$

with $s = s_{nb} - s_b$ and $\nu = \nu_+ + \nu_-$. While (7) does not conserve the total population size, the temporal evolution of x and of the total population size are decoupled from each other as a result of the deterministic dynamics (*1*).

Due to the environmental shifts depicted in Fig. 2a, $s(t)$ takes on a periodic form. The solution of (7) hence also approaches a periodic form, as detailed in (*1*) and also included in what follows. Starting with the neutral phase at $t = 0$, we have $s = 0$ in all four cases of Fig. 2c until $t = T_n$, hence the solution takes the form

$$x(t) = \frac{\nu_-}{\nu} + Ae^{-\nu t} \tag{8}$$

with the free parameter A. Following this, $s \neq 0$ in general during the selection period from $t = T_n$ until $t = T_n + T_s$, yielding

$$x(t) = \frac{1}{2s}\left(\tilde{s} - \hat{s}\frac{1 - Be^{-\hat{s}(t-T_n)}}{1 + Be^{-\hat{s}(t-T_n)}}\right) \tag{9}$$

with $\tilde{s} = s + \nu$, $\hat{s} = \sqrt{\tilde{s}^2 - 4s\nu_-}$ and the free parameter B. The value of A and B can be determined by imposing the boundary condition $x(0) = x(T)$ and the continuity constraint at $t = T_n$, yielding

$$\frac{\nu_-}{\nu} + A = \frac{1}{2s}\left(\tilde{s} - \hat{s}\frac{1 - Be^{-\hat{s}T_s}}{1 + Be^{-\hat{s}T_s}}\right), \qquad \frac{\nu_-}{\nu} + Ae^{-\nu T_n} = \frac{1}{2s}\left(\tilde{s} - \hat{s}\frac{1 - B}{1 + B}\right). \tag{10}$$

The constraints in (10) can be solved numerically to obtain the values of A and B, thus the expression of $x(t)$ from (8)–(9), which in turn yields the average fitness cost

$$\bar{s} = \frac{1}{T}\int_0^T \left[x(t)s_{nb}(t) + (1 - x(t))\, s_b(t)\right] \mathrm{d}t. \tag{11}$$

Note that when $(\nu + s)T \ll 1$, we have $e^{-\nu t} \approx 1$ for $t \in [0, T_n]$ and $e^{-\hat{s}(t-T_n)} \approx 1$ for $t \in [T_n, T]$, hence during both the neutral and the selection phase the solution in (8) remains approximately constant. As a result, the fraction $x(t)$ of non-binders remains approximately constant during the entire period due to the boundary and continuity conditions in this special case.

In Supplementary Fig. 4–Supplementary Fig. 15 we present detailed simulation data complementing the results featured in Fig. 3, indicating the dominant regulatory scheme(s) first for only non-autoregulated control (in Supplementary Fig. 4–Supplementary Fig. 9), then also including autoregulation (in Supplementary Fig. 10–Supplementary Fig. 15) considering low, medium, and high mutation rates in the presence of both weak and strong selection.

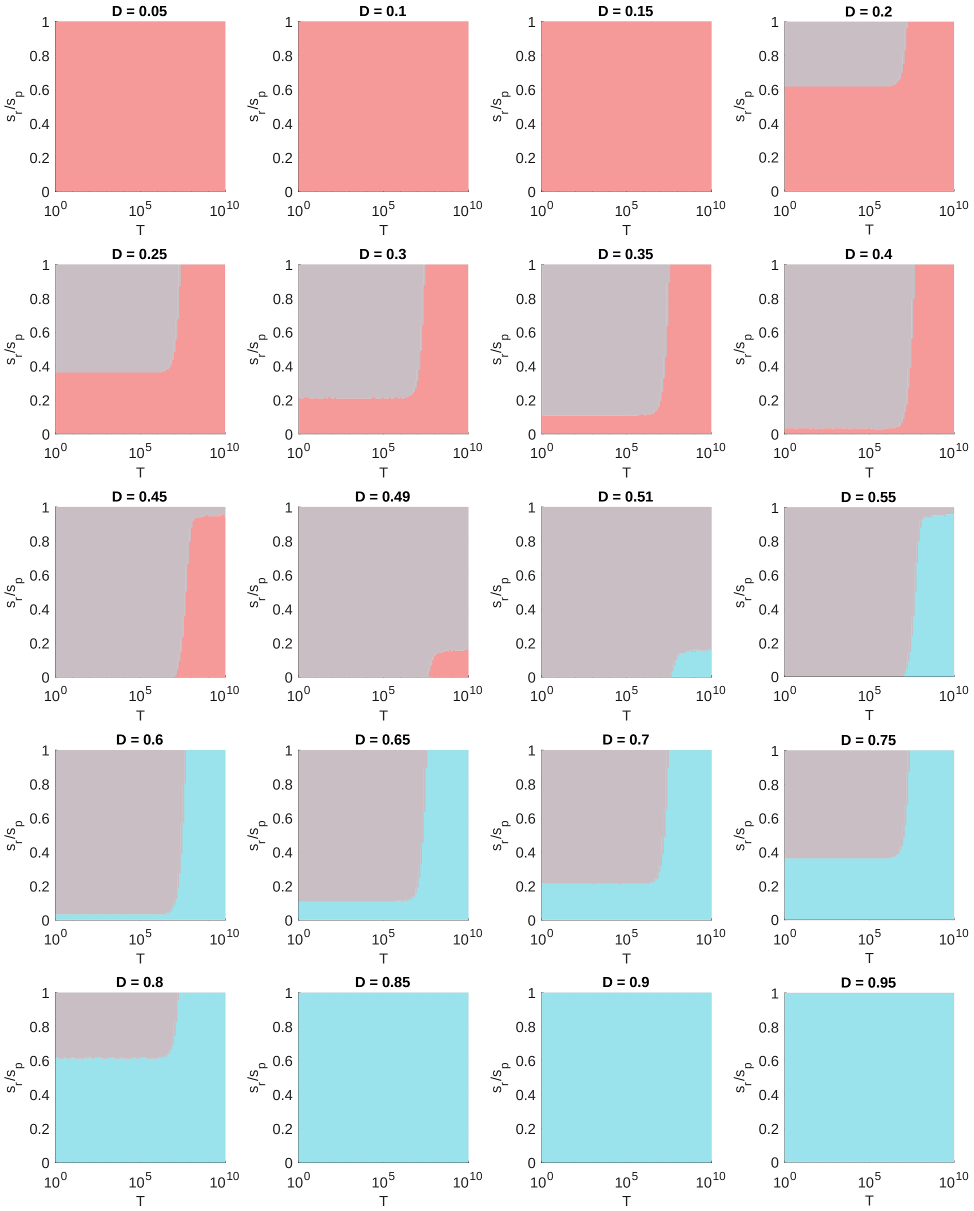

Supplementary Fig. 4. **Dominant regulatory scheme considering only non-autoregulated control (low mutation rate, weak selection).** Light red/blue denotes regions where non-autoregulated activation/repression dominates the other with at least 1% difference in the average fitness cost $\bar{s}$, in the light gray region the difference is less than 1%. Simulation parameters are: $\nu_- = 10^{-7}$, $\nu_+ = \nu_-/10$, $s_p = 10\nu_-$. The demand D is indicated above each panel.

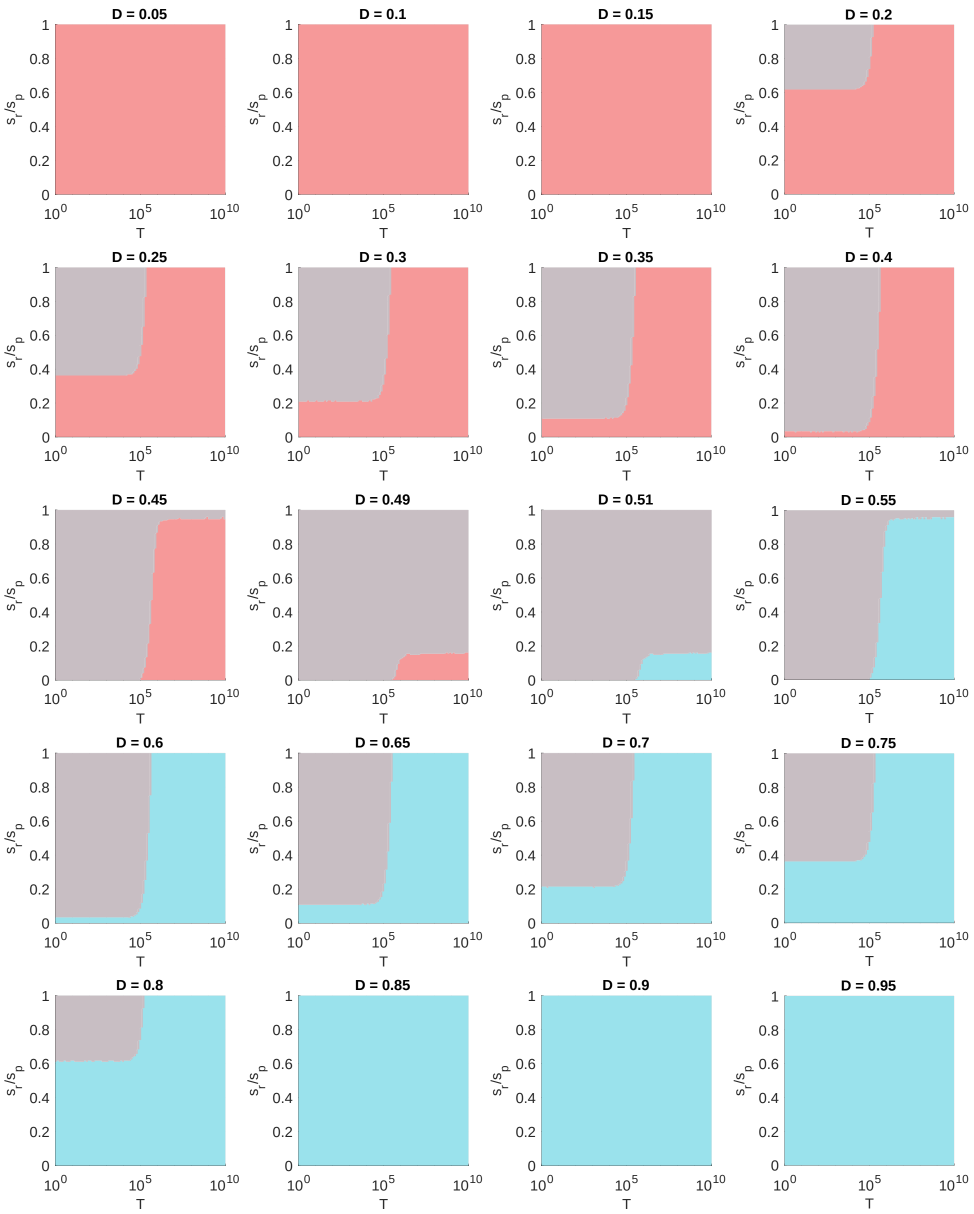

Supplementary Fig. 5. **Dominant regulatory scheme considering only non-autoregulated control (medium mutation rate, weak selection).** Light red/blue denotes regions where non-autoregulated activation/repression dominates the other with at least 1% difference in the average fitness cost $\bar{s}$, in the light gray region the difference is less than 1%. Simulation parameters are: $\nu_- = 10^{-5}$, $\nu_+ = \nu_-/10$, $s_p = 10\nu_-$. The demand D is indicated above each panel.

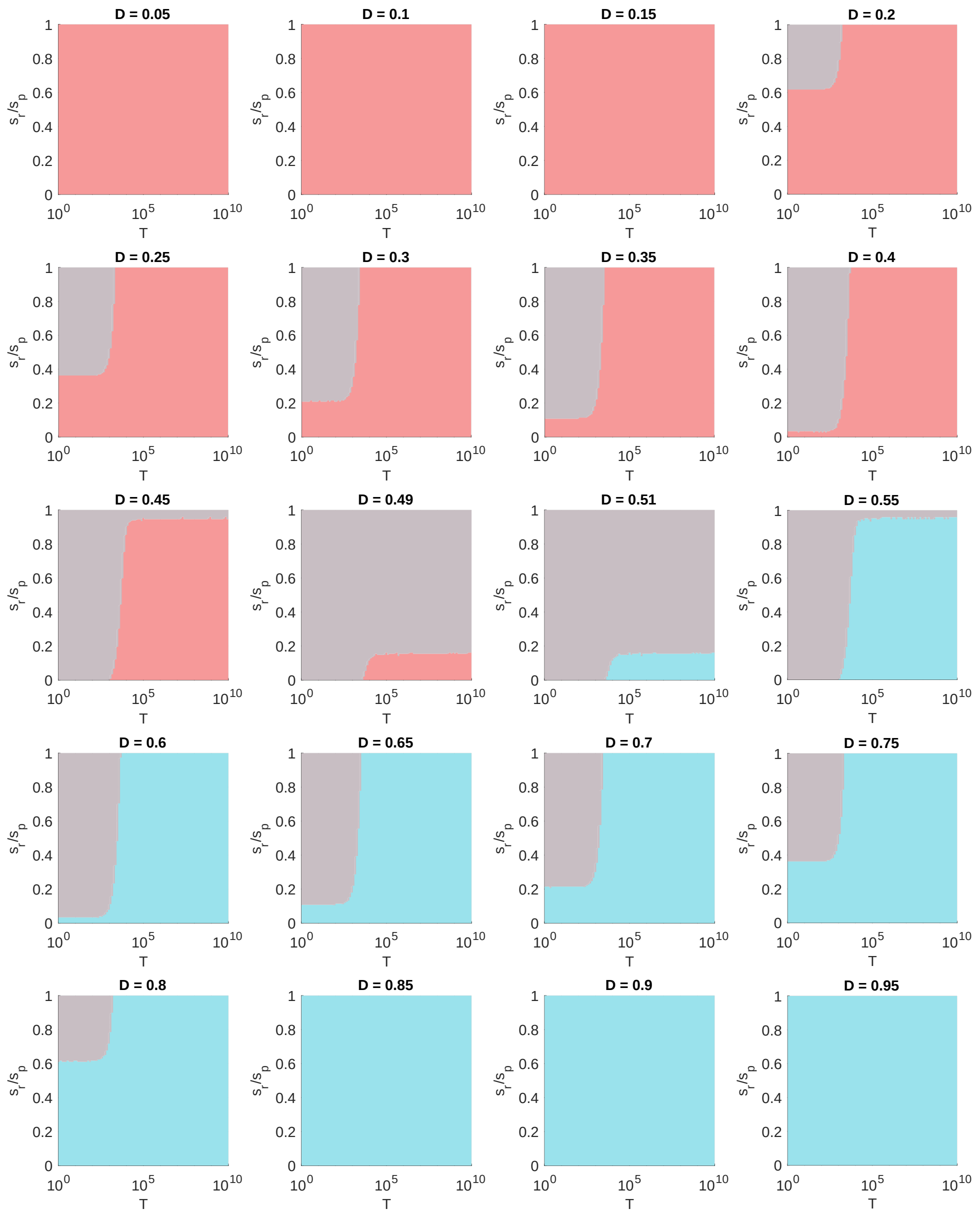

Supplementary Fig. 6. **Dominant regulatory scheme considering only non-autoregulated control (high mutation rate, weak selection).** Light red/blue denotes regions where non-autoregulated activation/repression dominates the other with at least 1% difference in the average fitness cost $\bar{s}$, in the light gray region the difference is less than 1%. Simulation parameters are: $\nu_- = 10^{-3}$, $\nu_+ = \nu_-/10$, $s_p = 10\nu_-$. The demand D is indicated above each panel.

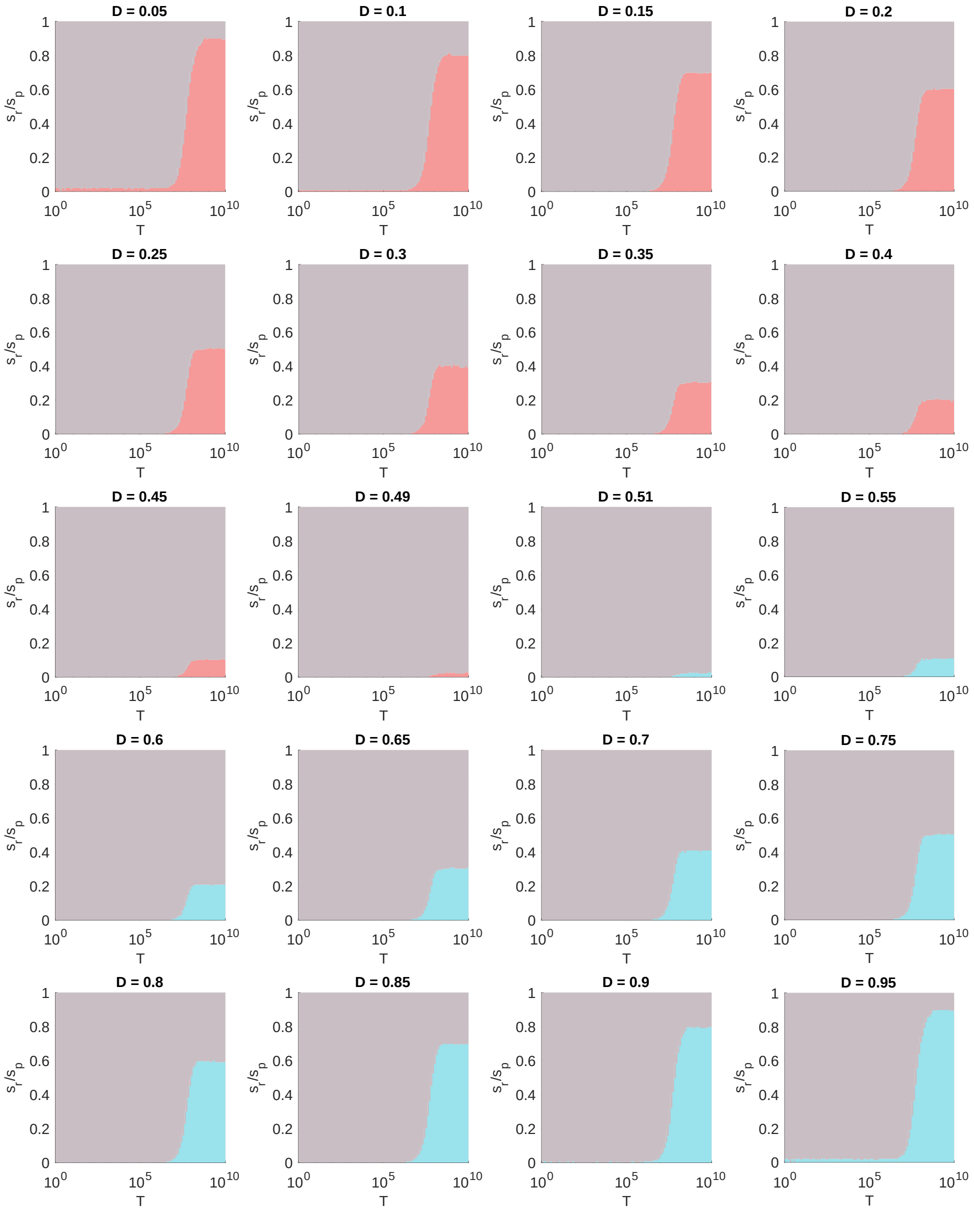

Supplementary Fig. 7. **Dominant regulatory scheme considering only non-autoregulated control (low mutation rate, strong selection).** Light red/blue denotes regions where non-autoregulated activation/repression dominates the other with at least 1% difference in the average fitness cost $\bar{s}$, in the light gray region the difference is less than 1%. Simulation parameters are: $\nu_- = 10^{-7}$, $\nu_+ = \nu_-/10$, $s_p = 100\nu_-$. The demand D is indicated above each panel.

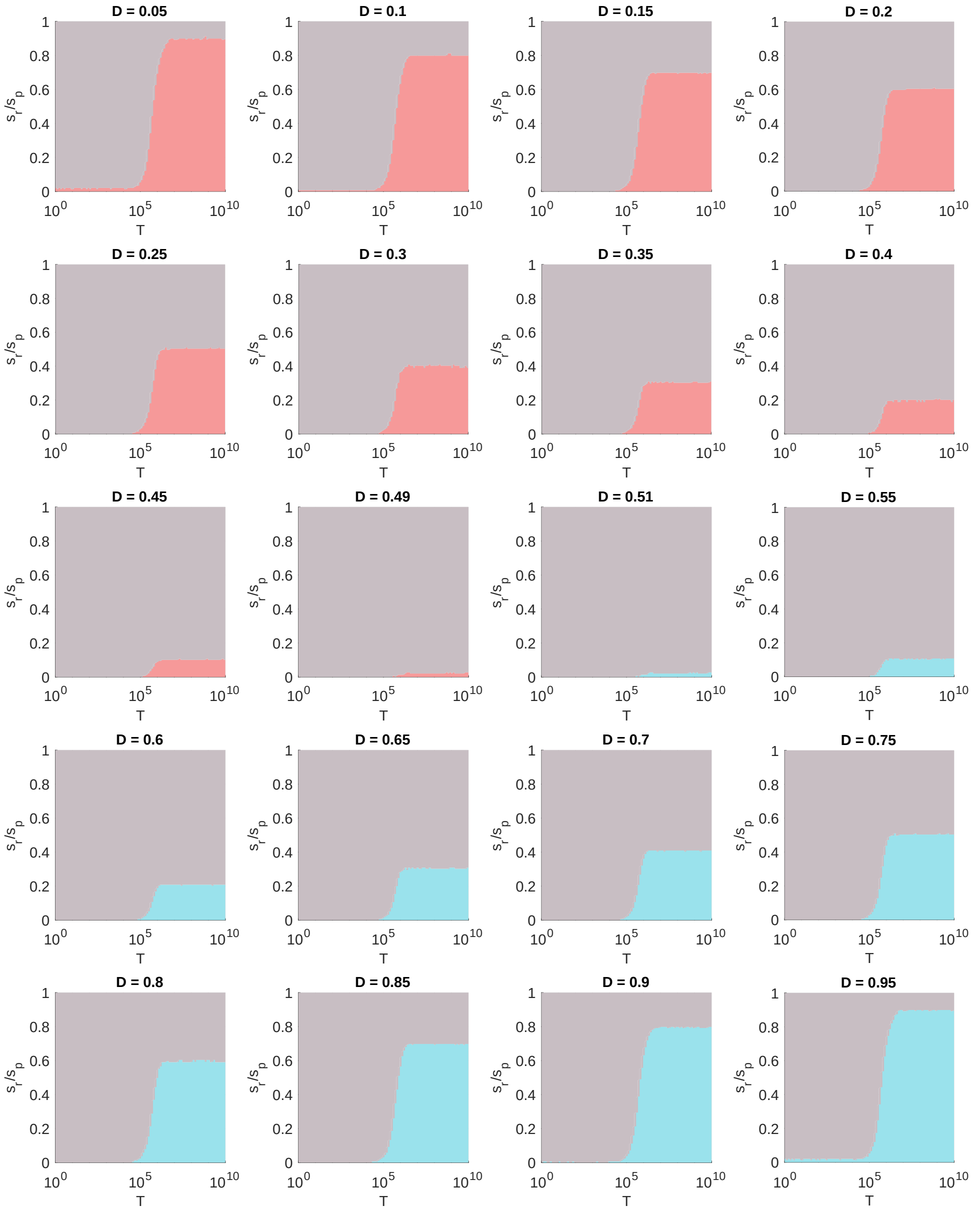

Supplementary Fig. 8. **Dominant regulatory scheme considering only non-autoregulated control (medium mutation rate, strong selection).** Light red/blue denotes regions where non-autoregulated activation/repression dominates the other with at least 1% difference in the average fitness cost $\bar{s}$, in the light gray region the difference is less than 1%. Simulation parameters are: $\nu_- = 10^{-5}$, $\nu_+ = \nu_-/10$, $s_p = 100\nu_-$. The demand D is indicated above each panel.

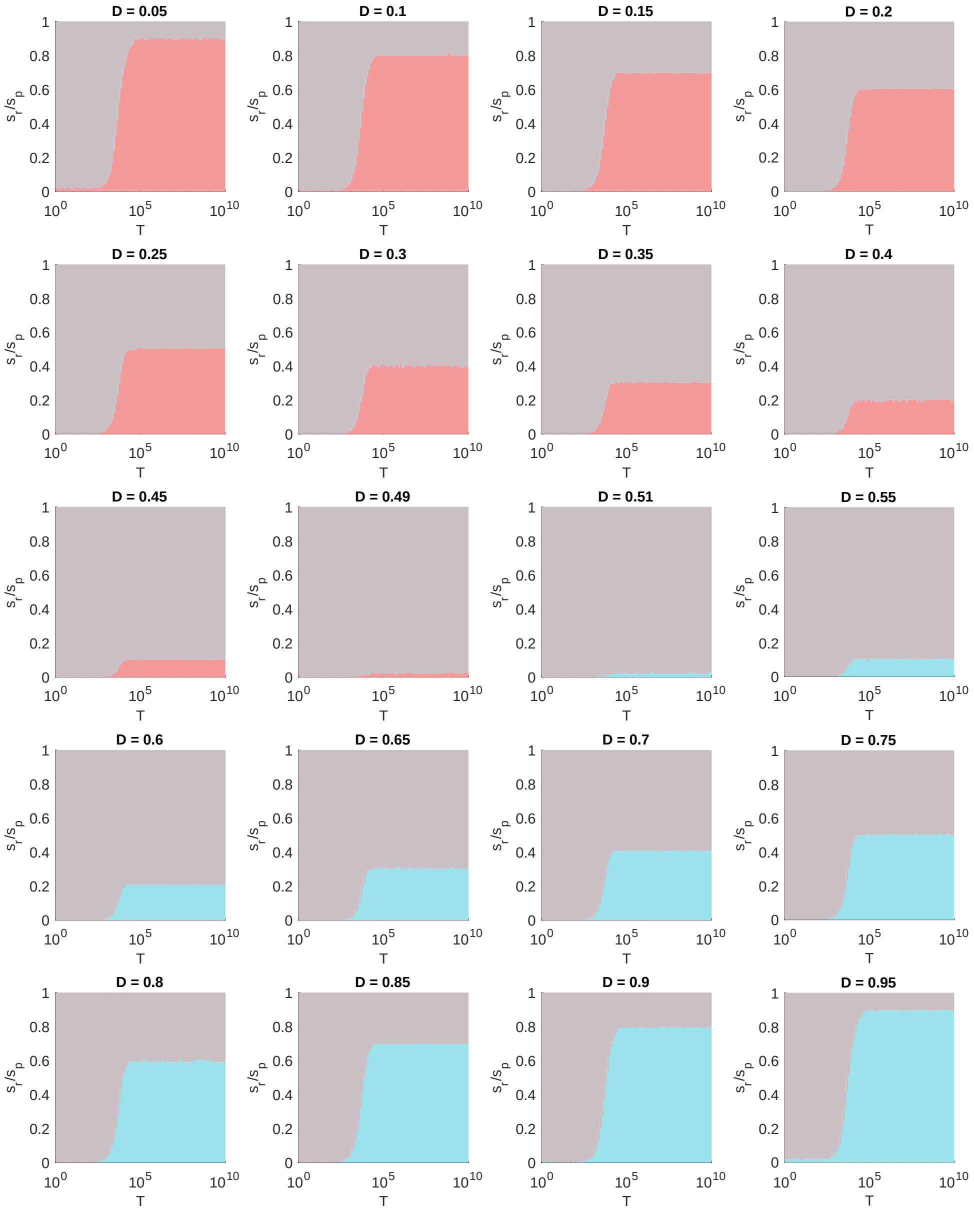

Supplementary Fig. 9. **Dominant regulatory scheme considering only non-autoregulated control (high mutation rate, strong selection).** Light red/blue denotes regions where non-autoregulated activation/repression dominates the other with at least 1% difference in the average fitness cost $\bar{s}$, in the light gray region the difference is less than 1%. Simulation parameters are: $\nu_- = 10^{-3}$, $\nu_+ = \nu_-/10$, $s_p = 100\nu_-$. The demand D is indicated above each panel.

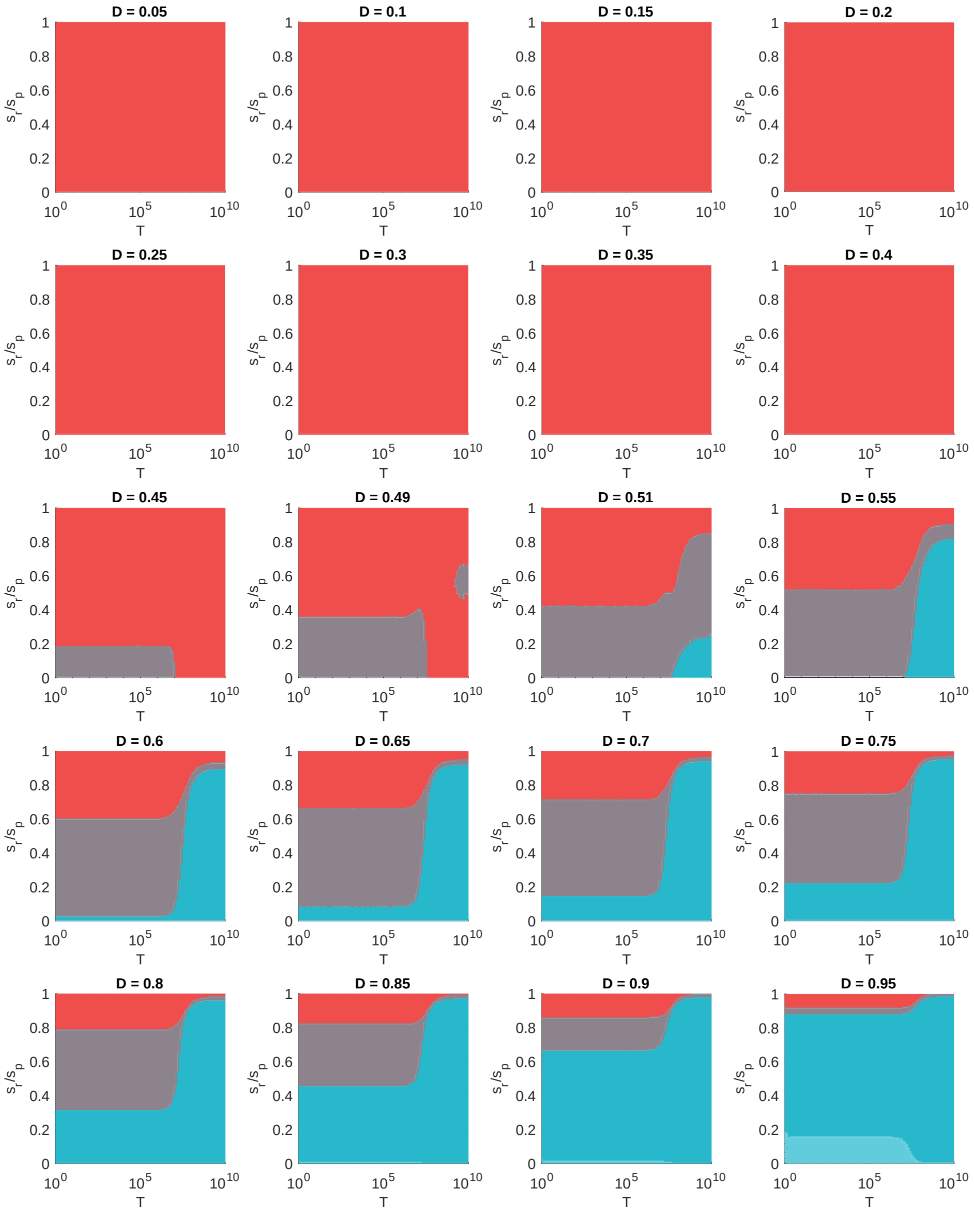

Supplementary Fig. 10. **Dominant regulatory scheme considering both non-autoregulated and autoregulated control (low mutation rate, weak selection).** Colors indicate the control scheme(s) with the lowest fitness cost $\bar{s}$: in case of a single winner, the second best is dominated by at least 1%; in case of multiple winners, their difference is less than 1% and all other variants are at least 1% worse. Dark red/blue indicates regions where autoregulated activation/repression dominates, light blue corresponds to regions where non-autoregulated and autoregulated repression dominate non-autoregulated and autoregulated activation, in the dark gray region autoregulated activation and autoregulated repression have comparable performance dominating their non-autoregulated counterparts. Simulation parameters are: $\nu_- = 10^{-7}$, $\nu_+ = \nu_-/10$, $s_p = 10\nu_-$. The demand D is indicated above each panel.

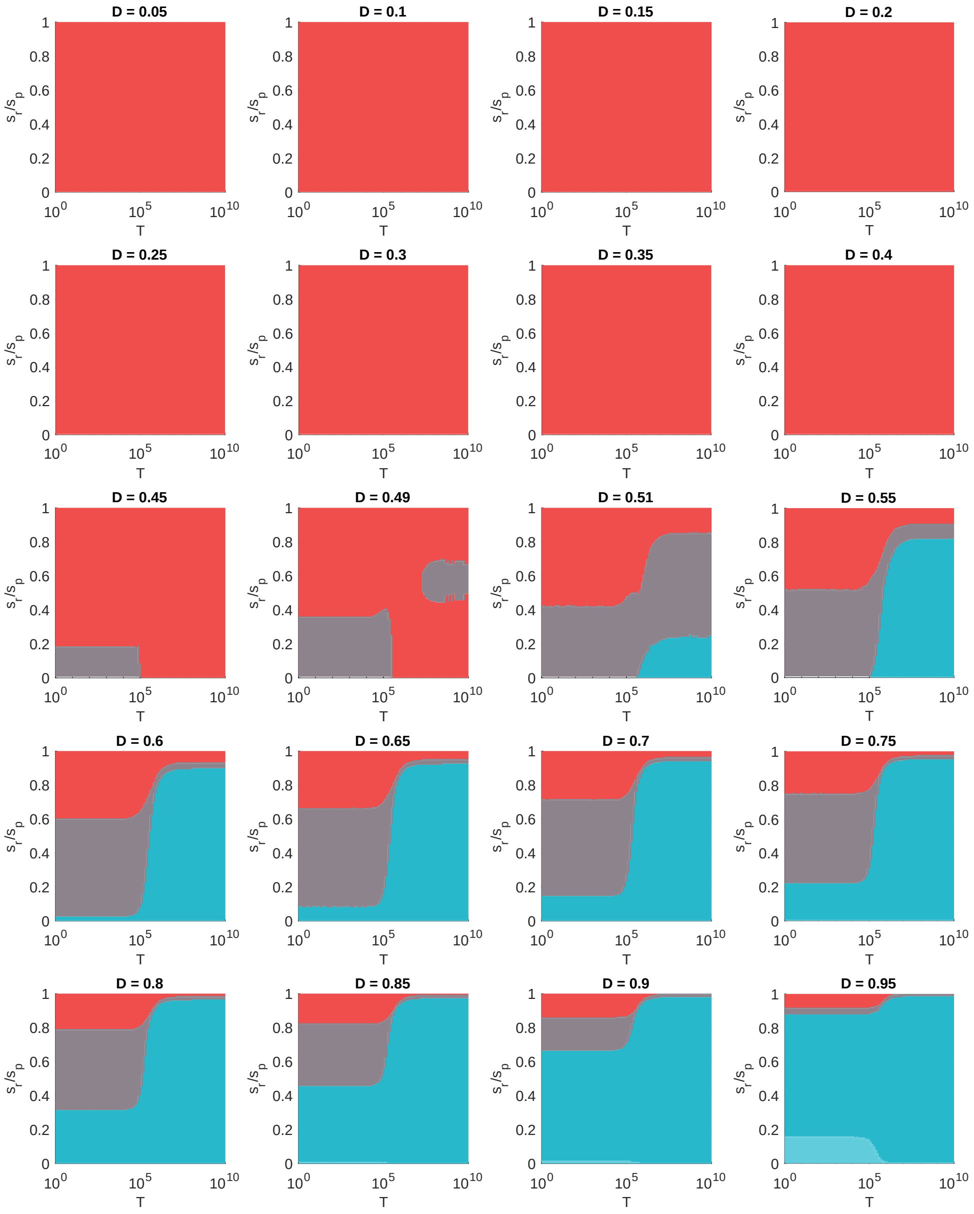

Supplementary Fig. 11. **Dominant regulatory scheme considering both non-autoregulated and autoregulated control (medium mutation rate, weak selection).** Colors indicate the control scheme(s) with the lowest fitness cost $\bar{s}$: in case of a single winner, the second best is dominated by at least 1%; in case of multiple winners, their difference is less than 1% and all other variants are at least 1% worse. Dark red/blue indicates regions where autoregulated activation/repression dominates, light blue corresponds to regions where non-autoregulated and autoregulated repression dominate non-autoregulated and autoregulated activation, in the dark gray region autoregulated activation and autoregulated repression have comparable performance dominating their non-autoregulated counterparts. Simulation parameters are: $\nu_- = 10^{-5}$, $\nu_+ = \nu_-/10$, $s_p = 10\nu_-$. The demand D is indicated above each panel.

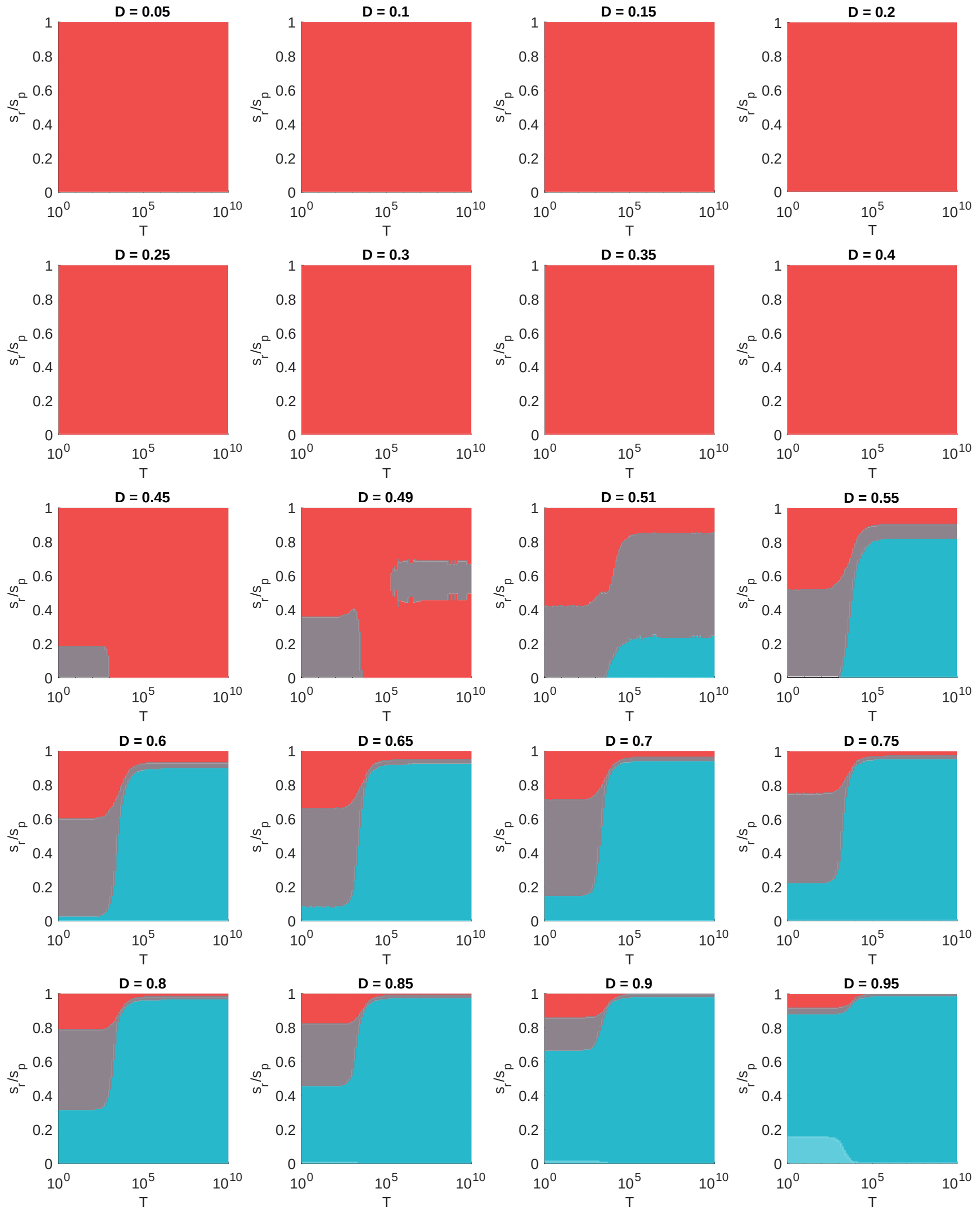

Supplementary Fig. 12. **Dominant regulatory scheme considering both non-autoregulated and autoregulated control (high mutation rate, weak selection).** Colors indicate the control scheme(s) with the lowest fitness cost $\bar{s}$: in case of a single winner, the second best is dominated by at least 1%; in case of multiple winners, their difference is less than 1% and all other variants are at least 1% worse. Dark red/blue indicates regions where autoregulated activation/repression dominates, light blue corresponds to regions where non-autoregulated and autoregulated repression dominate non-autoregulated and autoregulated activation, in the dark gray region autoregulated activation and autoregulated repression have comparable performance dominating their non-autoregulated counterparts. Simulation parameters are: $\nu_- = 10^{-3}$, $\nu_+ = \nu_-/10$, $s_p = 10\nu_-$. The demand D is indicated above each panel.

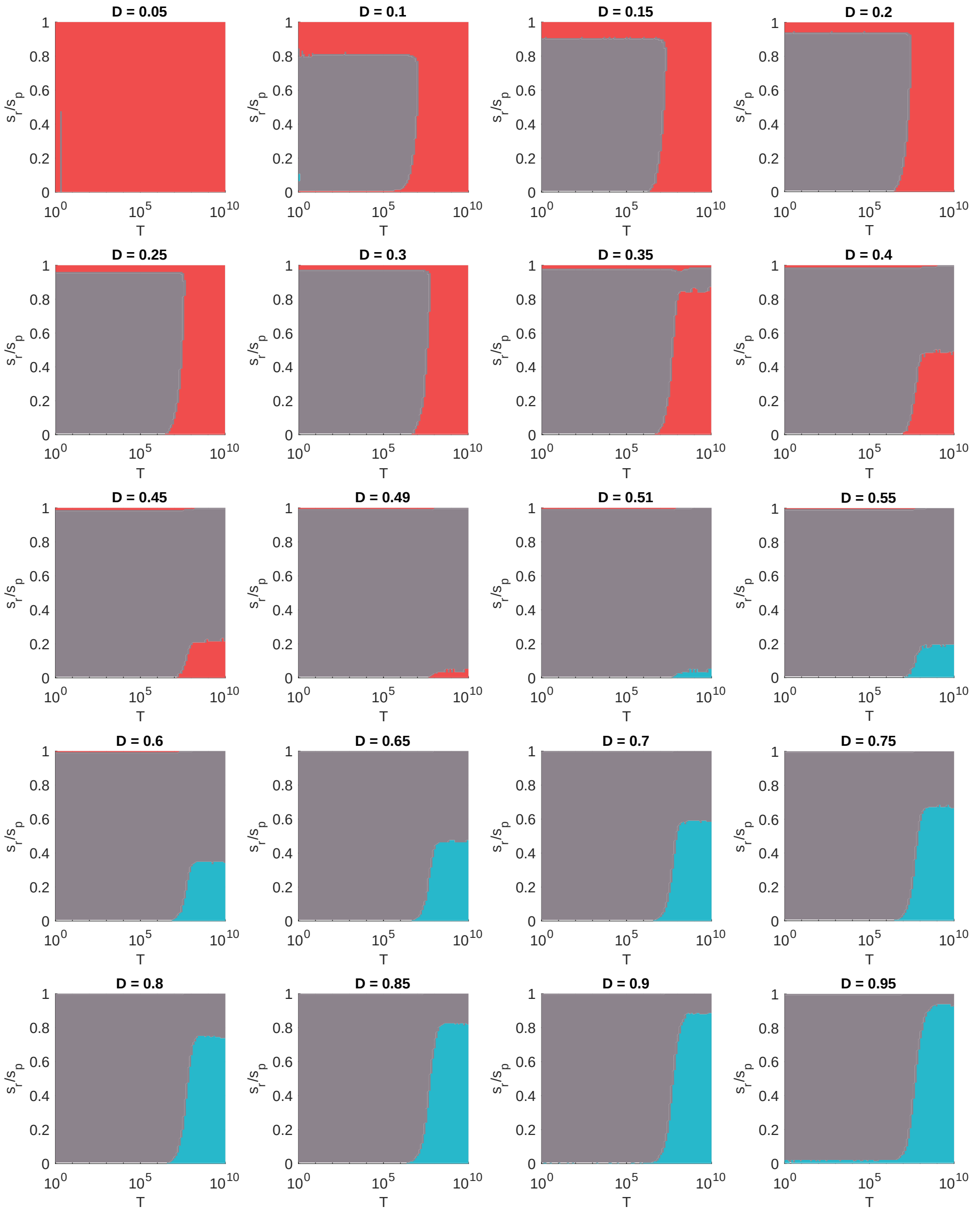

Supplementary Fig. 13. **Dominant regulatory scheme considering both non-autoregulated and autoregulated control (low mutation rate, strong selection).** Colors indicate the control scheme(s) with the lowest fitness cost $\bar{s}$: in case of a single winner, the second best is dominated by at least 1%; in case of multiple winners, their difference is less than 1% and all other variants are at least 1% worse. Dark red/blue indicates regions where autoregulated activation/repression dominates, light blue corresponds to regions where non-autoregulated and autoregulated repression dominate non-autoregulated and autoregulated activation, in the dark gray region autoregulated activation and autoregulated repression have comparable performance dominating their non-autoregulated counterparts. Simulation parameters are: $\nu_- = 10^{-7}$, $\nu_+ = \nu_-/10$, $s_p = 100\nu_-$. The demand D is indicated above each panel.

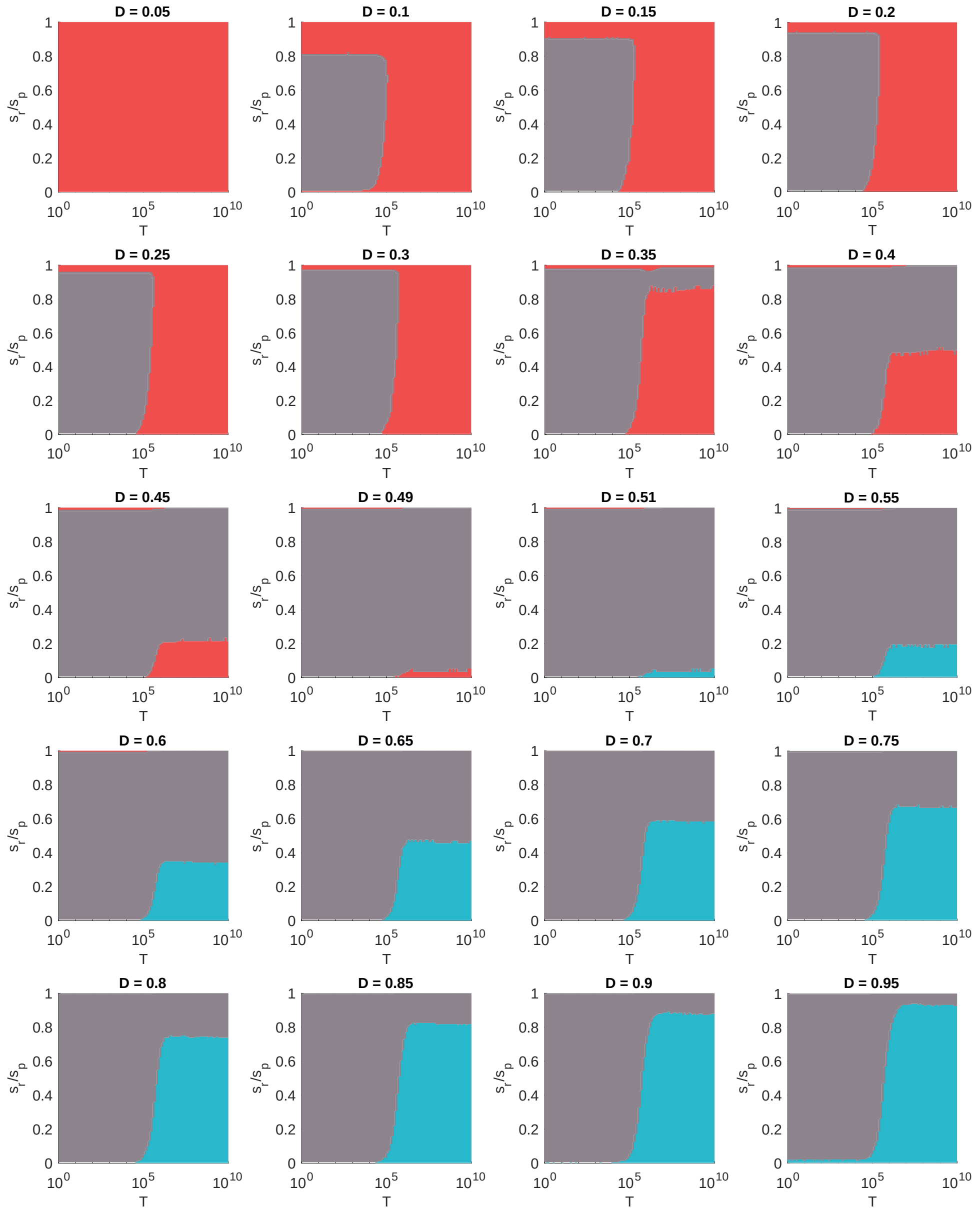

Supplementary Fig. 14. **Dominant regulatory scheme considering both non-autoregulated and autoregulated control (medium mutation rate, strong selection).** Colors indicate the control scheme(s) with the lowest fitness cost $\bar{s}$: in case of a single winner, the second best is dominated by at least 1%; in case of multiple winners, their difference is less than 1% and all other variants are at least 1% worse. Dark red/blue indicates regions where autoregulated activation/repression dominates, light blue corresponds to regions where non-autoregulated and autoregulated repression dominate non-autoregulated and autoregulated activation, in the dark gray region autoregulated activation and autoregulated repression have comparable performance dominating their non-autoregulated counterparts. Simulation parameters are: $\nu_- = 10^{-5}$, $\nu_+ = \nu_-/10$, $s_p = 100\nu_-$. The demand D is indicated above each panel.

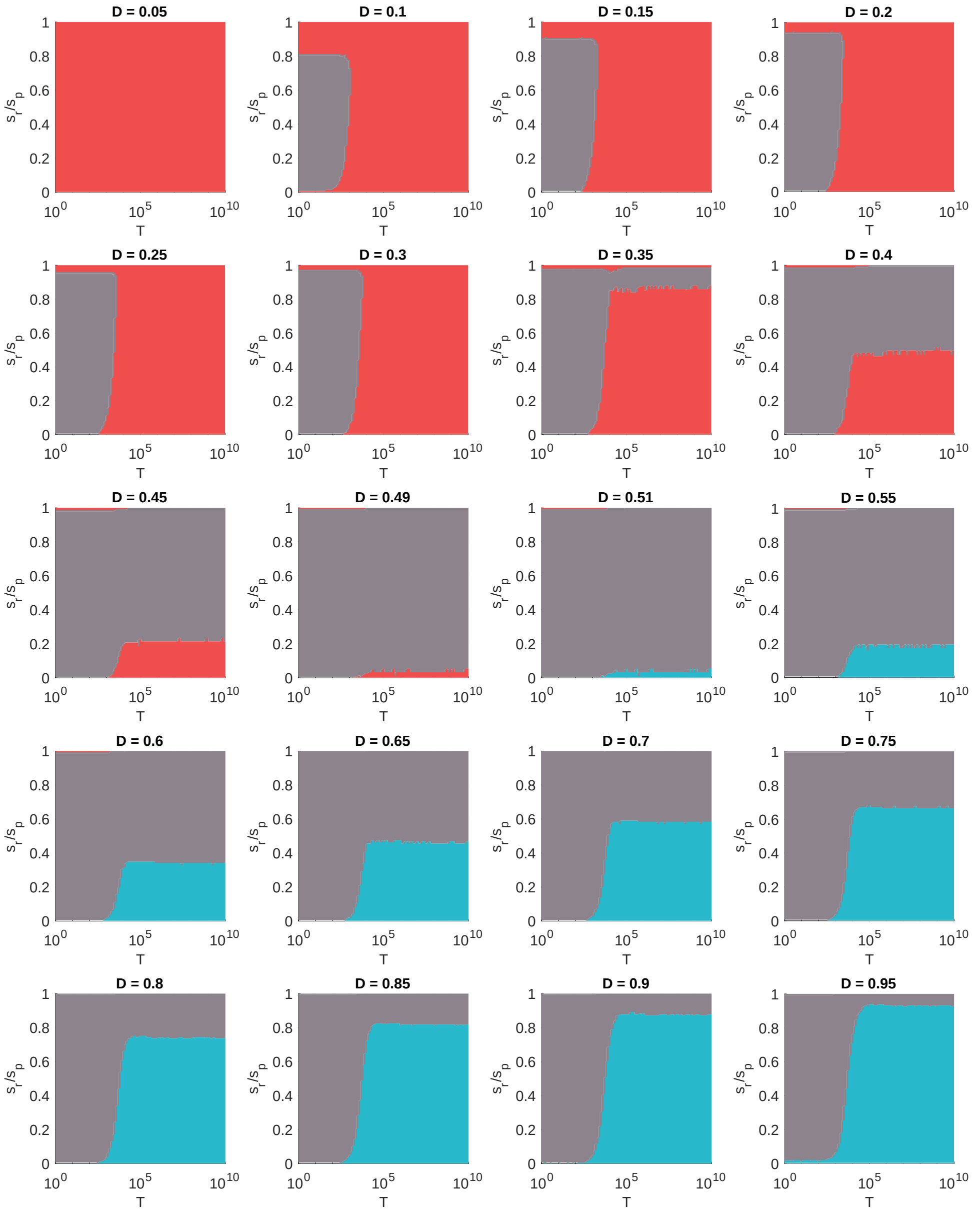

Supplementary Fig. 15. **Dominant regulatory scheme considering both non-autoregulated and autoregulated control (high mutation rate, strong selection).** Colors indicate the control scheme(s) with the lowest fitness cost $\bar{s}$: in case of a single winner, the second best is dominated by at least 1%; in case of multiple winners, their difference is less than 1% and all other variants are at least 1% worse. Dark red/blue indicates regions where autoregulated activation/repression dominates, light blue corresponds to regions where non-autoregulated and autoregulated repression dominate non-autoregulated and autoregulated activation, in the dark gray region autoregulated activation and autoregulated repression have comparable performance dominating their non-autoregulated counterparts. Simulation parameters are: $\nu_- = 10^{-3}$, $\nu_+ = \nu_-/10$, $s_p = 100\nu_-$. The demand D is indicated above each panel.

3 Fitness cost in small populations

The stochastic sampling algorithm ("Stochastic simulation of the evolutionary dynamics" in the Methods) relies on a large number of computational steps, and thus often requires a prohibitively long time to complete (e.g., approximately 35 hours for Fig. 4a). Data presented in Supplementary Fig. 16 demonstrate that relying on the diffusion approximation ("Periodic steady state distribution" in the Methods) does not compromise the results we obtain considering a variety of scenarios and parameter combinations, while offering a dramatic decrease in computational time (approximately 100-fold). Following this, in Supplementary Fig. 17a we verify that the stochastic sampling algorithm we rely on produces results identical to those originally featured in (*1*). Additionally, in Supplementary Fig. 17b we include detailed numerical simulations complementing data presented in Fig. 4a to illustrate that the dominant non-autoregulated regulatory scheme is generally in accordance with wear-and-tear principle, and the use-it-or-lose-it principle emerges only under certain circumstances in small populations.

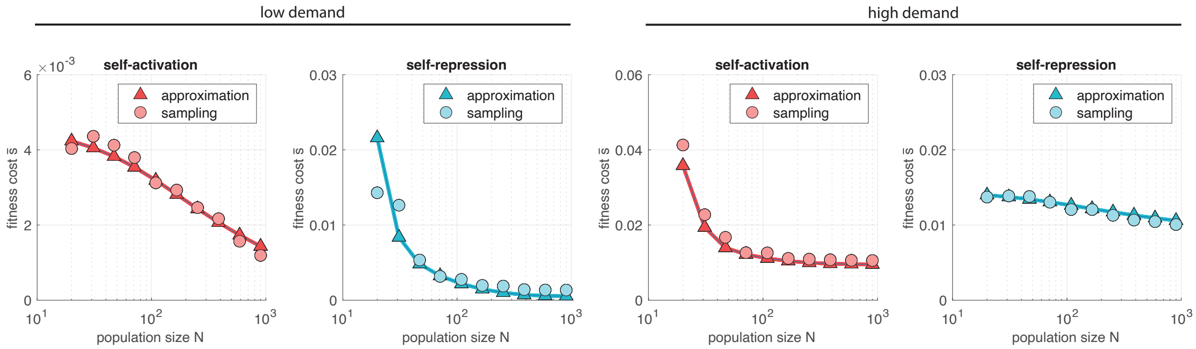

Supplementary Fig. 16. **Validation of the diffusion approximation.** Data are obtained using the stochastic sampling algorithm (circles) and its diffusion approximation (triangles) described in the Methods. In all plots $\nu_- = 10^{-3}$, $\nu_+ = \nu_-/10$, $T = 10^4$, $s_p = 100\nu_-$, $s_r = s_p/10$. Low demand and high demand refers to $D = 0.05$ and $D = 0.95$, respectively. For stochastic sampling, results are averaged over 10 replicates with 10 cycles each, starting from the initial point $x = 0.5$. For the diffusion approximation, 30 spatial and 1000 temporal points are considered when solving the PDE with a uniform initial distribution.

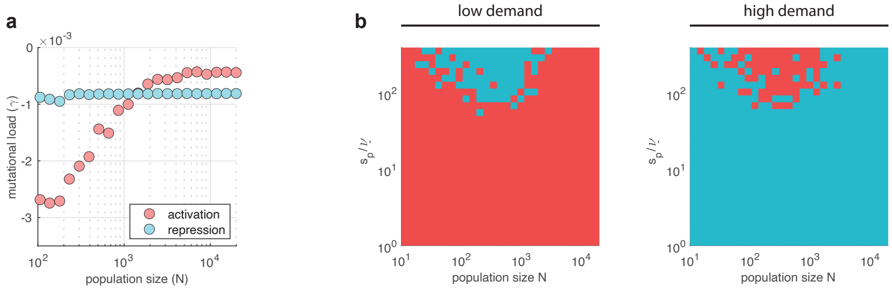

Supplementary Fig. 17. **Detailed numerical simulations complementing Fig. 4a.** Red and blue corresponds to non-autoregulated activation and repression, respectively. Data are obtained using the stochastic sampling algorithm described in the Methods, averaged over 10 periods and 10 replicates with initial condition $N_b = N_{nb} = N/2$. Simulation parameters are: $\nu_- = 10^{-3}$, $\nu_+ = \nu_-/10$, $s_p = 0.2$. Low and high demand refers to $T_i = 100$, $T_{ni} = 5000$ and $T_i = 5000$, $T_{ni} = 100$, respectively. **a** Replicating the data in Fig. 3B of (*1*) in the low demand case with the original notation $\gamma = -\bar{s}$ representing the mutational load. **b** Red and blue indicates where non-autoregulated activation and repression dominates, respectively.

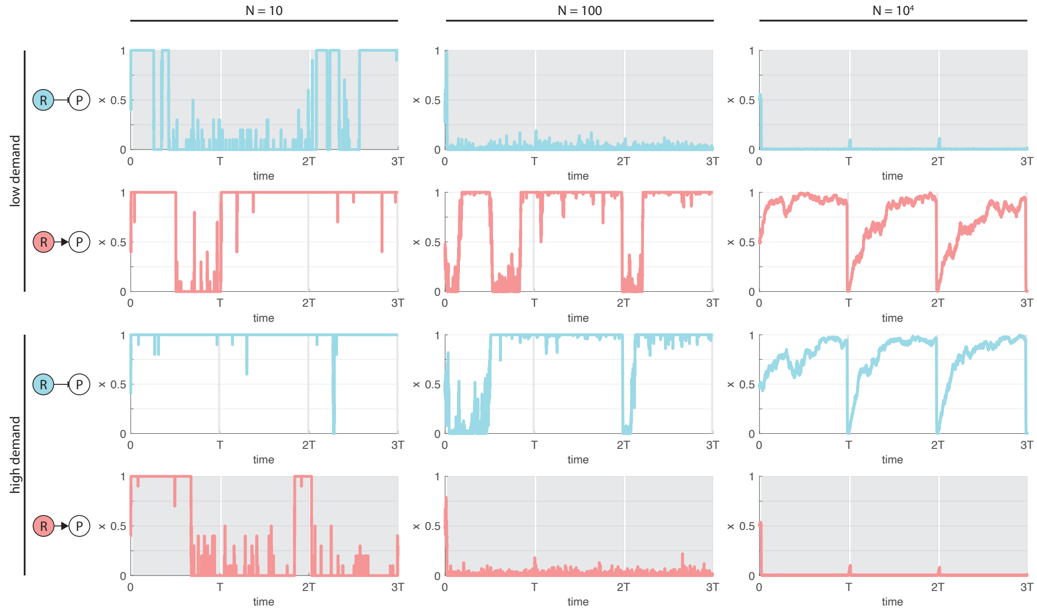

Supplementary Fig. 18. **Impact of population size on rare stochastic events.** Data are obtained using the sampling algorithm described in the Methods ("Stochastic simulation of the evolutionary dynamics") with initial condition $N_b = N_{nb} = N/2$. Gray regions denote the selection phase. Low and high demand refers to $D = 0.02$ and $D = 0.98$, respectively. Simulation parameters are: $\nu_- = 10^{-3}$, $\nu_+ = \nu_-/10$, $s_p = 0.2$, $T = 5000$.

In Supplementary Fig. 18 we highlight how population size affects the fraction of non-binders in case of non-autoregulated control. In Supplementary Fig. 19–Supplementary Fig. 20 we illustrate the impact of selection pressure on rare stochastic events to reveal that increasing s_p drastically reduces the frequency of non-binders taking over the population in case of activation/repression in high/low demand scenarios. This phenomenon holds for both non-autoregulated and autoregulated control. Finally, using the color scheme in Supplementary Table 1, in Supplementary Fig. 21 we present detailed simulation data complementing the results featured in Fig. 4b.

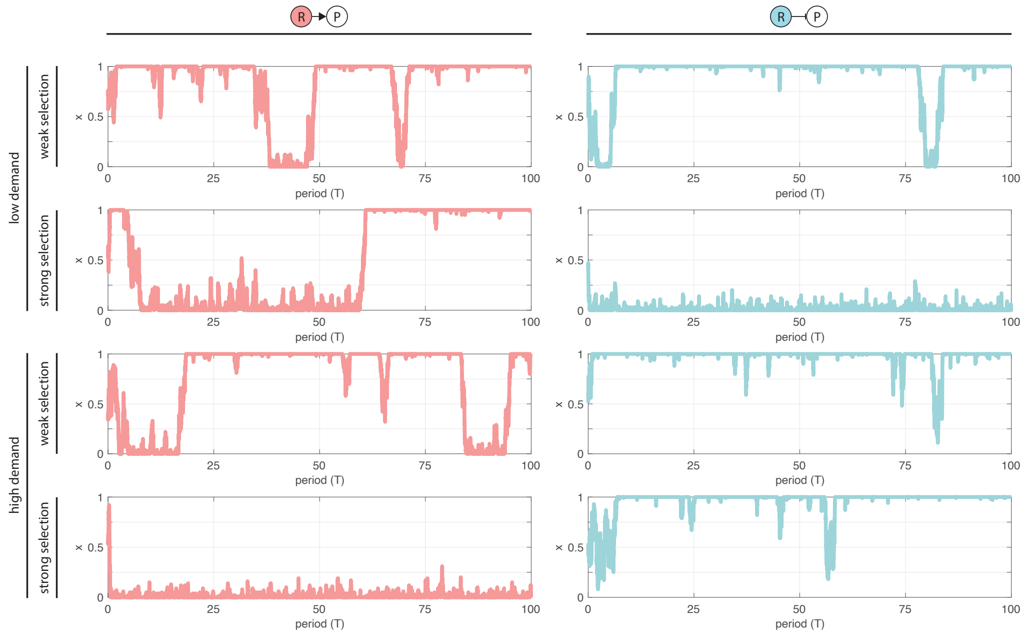

Supplementary Fig. 19. **Impact of selection pressure on rare stochastic events (non-autoregulated control).** Data are obtained using the sampling algorithm described in the Methods (“Stochastic simulation of the evolutionary dynamics”) with initial condition $N_b = N_{nb} = N/2$. Low and high demand refers to $D = 0.02$ and $D = 0.98$, respectively. Weak and strong selection corresponds to $s_p = 10\nu_-$ and $s_p = 100\nu_-$, respectively. Simulation parameters are: $\nu_- = 10^{-3}$, $\nu_+ = \nu_-/10$, $T = 100$, $N = 100$.

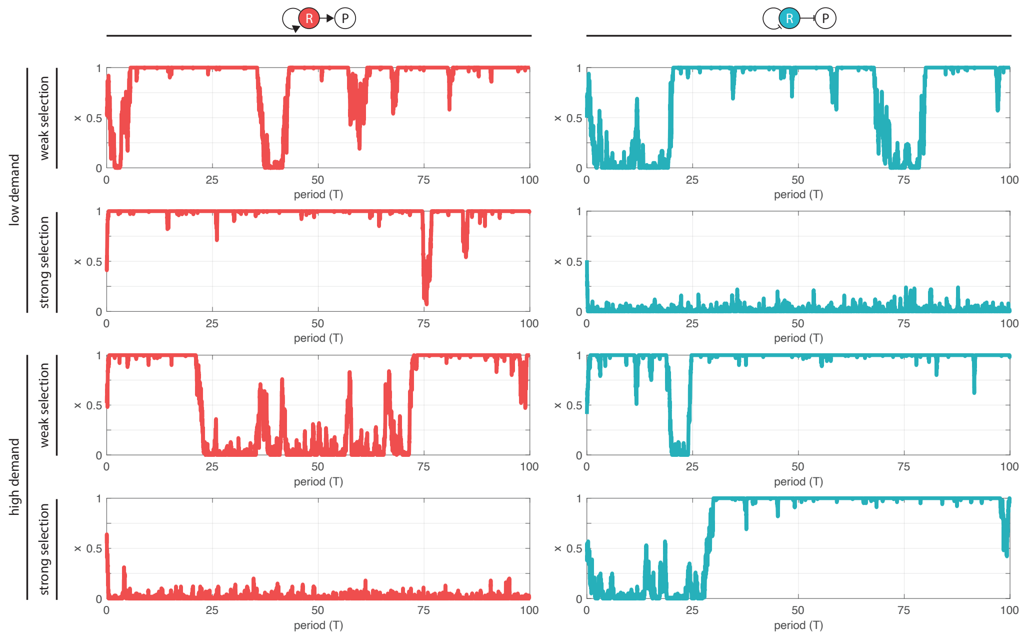

Supplementary Fig. 20. **Impact of selection pressure on rare stochastic events (autoregulation).** Data are obtained using the sampling algorithm described in the Methods ("Stochastic simulation of the evolutionary dynamics") with initial condition $N_b = N_{nb} = N/2$. Low and high demand refers to $D = 0.02$ and $D = 0.98$, respectively. Weak and strong selection corresponds to $s_p = 10\nu_-$ and $s_p = 100\nu_-$, respectively. Simulation parameters are: $\nu_- = 10^{-3}$, $\nu_+ = \nu_-/10$, $T = 100$, $N = 100$.

Supplementary Table 1. **Color scheme for indicating dominant strategies.** Dominant strategies with the lowest fitness cost $\bar{s}$ are denoted by "x" in each column. In case of a single winner, the second best is dominated by at least 1%; in case of multiple winners, their difference is less than 1% and all other variants are at least 1% worse.

non-autoreg. activation	x		x		x		x		x		x		x		x
non-autoreg. repression		x	x			x	x			x	x			x	x
autoregulated activation				x	x	x	x					x	x	x	x
autoregulated repression								x	x	x	x	x	x	x	x

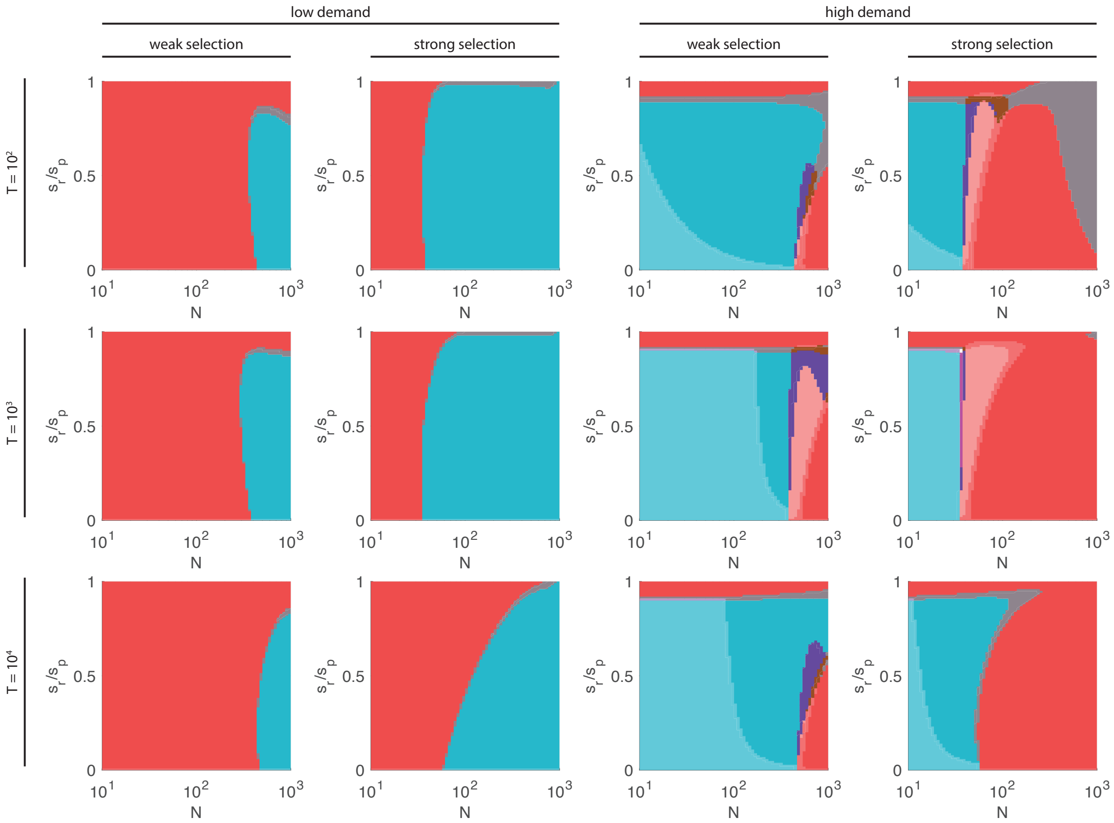

Supplementary Fig. 21. **Evolutionary advantageous regulatory motifs based on fitness cost in small populations.** Colors from Supplementary Table 1 indicate the control scheme(s) with the lowest fitness cost $\bar{s}$: in case of a single winner, the second best is dominated by at least 1%; in case of multiple winners, their difference is less than 1% and all other variants are at least 1% worse. Data are obtained using the diffusion approximation described in the Methods. Low and high demand refers to $D = 0.05$ and $D = 0.95$, respectively. Weak and strong selection corresponds to $s_p = 10\nu_-$ and $s_p = 100\nu_-$, respectively. Simulation parameters are: $\nu_- = 10^{-3}$, $\nu_+ = \nu_-/10$.

4 Autoregulation can result in unwanted selection pressure

In Supplementary Fig. 22–Supplementary Fig. 23, we present simulation data (from the stochastic sampling algorithm) complementing the results featured in Fig. 5 to illustrate that while increasing the R-cost via s_r shifts the distribution towards non-binders in case of self-activation (Supplementary Fig. 22) as it decreases the selection pressure s in (7), for self-repression the shift is the exact opposite (Supplementary Fig. 23) due to increased selection pressure.

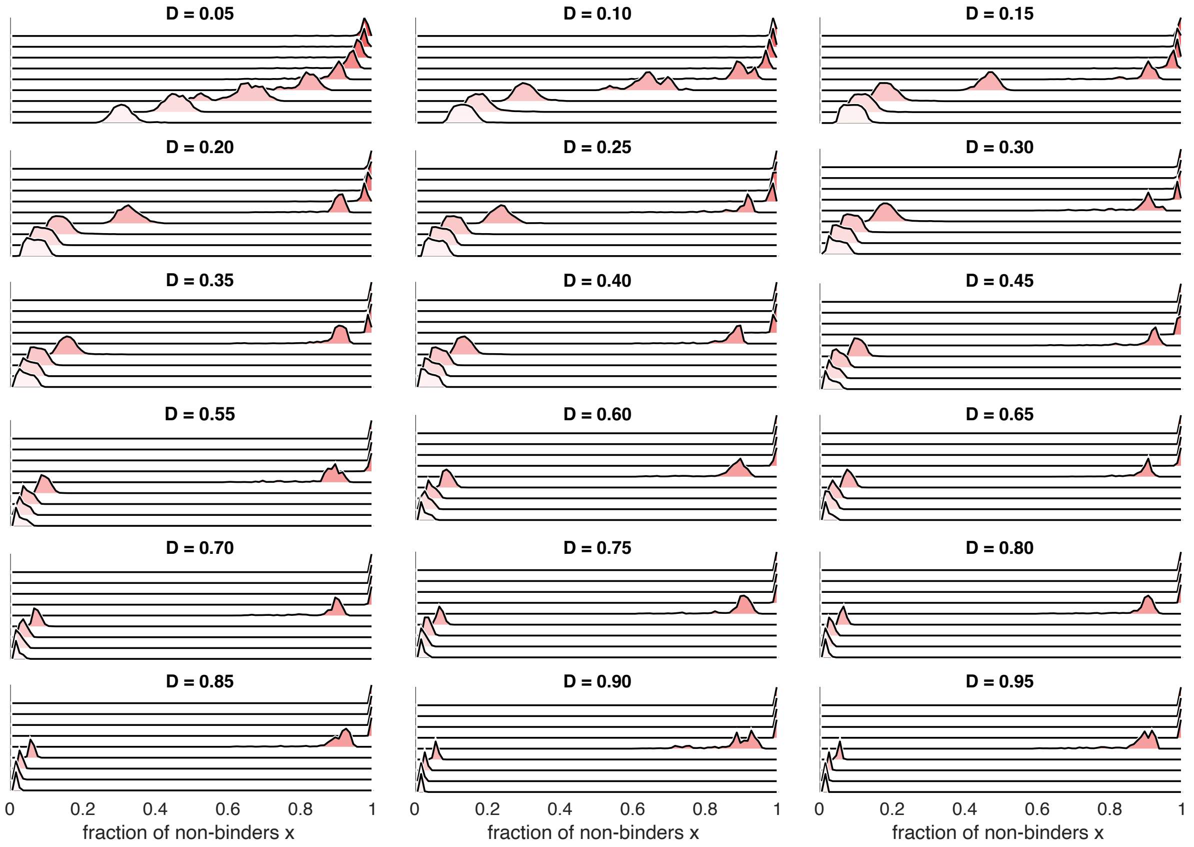

Supplementary Fig. 22. **Distribution of the fraction of non-binders considering self-activation of the regulator.** In all plots $\nu_- = 10^{-3}$, $\nu_+ = \nu_-/10$, $s_p = 100\nu_-$, $N = 1000$, $T = 100$. Results represent averages over 100 independent runs, each lasting 100 periods, where the first 3 periods are discarded to eliminate the effect of the initial condition ($x = 0.5$). Darker shades indicate greater values of s_r (ranging from $s_r = 0.1s_p$ to $s_r = 0.9s_p$). Distributions are visualized with identical maximal heights.

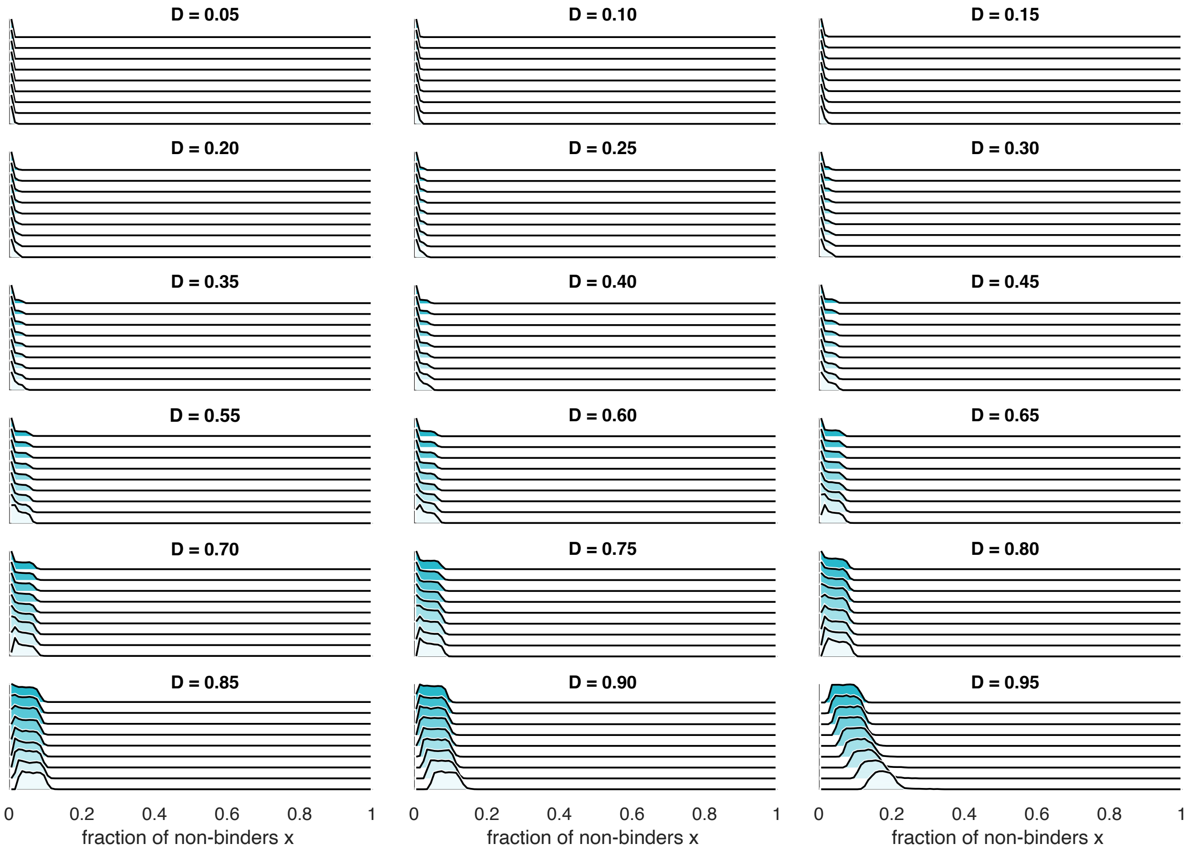

Supplementary Fig. 23. **Distribution of the fraction of non-binders considering self-repression of the regulator.** In all plots $\nu_- = 10^{-3}$, $\nu_+ = \nu_-/10$, $s_p = 100\nu_-$, $N = 1000$, $T = 100$. Results represent averages over 100 independent runs, each lasting 100 periods, where the first 3 periods are discarded to eliminate the effect of the initial condition ($x = 0.5$). Darker shades indicate greater values of s_r (ranging from $s_r = 0.1s_p$ to $s_r = 0.9s_p$.) Distributions are visualized with identical maximal heights.

In Supplementary Fig. 24 we reveal the role that the duration T of the period plays with respect to the critical timescales $T_b = 1/\nu_-$, $T_w = 1/(s_w + \nu_+)$, and $T^* = \max(T_b, T_w)$. When $T_w < T_b$, wipe-out happens faster than build-up, thus if $T < T^* = T_b$ then non-binders do not overtake the population during the neutral phase, hence $x \approx 0$ throughout the entire period, whereas once $T > T^* = T_b$, build-up and wipe-out alternate during the neutral and selection phases (Supplementary Fig. 24a). When $T_b < T_w$, build-up happens faster than wipe-out, thus always dominating it, resulting in $x \approx 1$ throughout the entire period (Supplementary Fig. 24b).

Considering the typical value of model parameters (see Methods), we have $s_p \gg \nu_-$. As a result, for non-autoregulated activation and for non-autoregulated/autoregulated repression we have $T^* = T_b$. For autoregulated activation, we also have $T^* = T_b$ as long as $s_r < s_p + \nu_- - \nu_+$ (which is the case considering the typical value of parameters), otherwise $T^* = T_w$. Since loss-of-function mutations are assumed to be more frequent than gain-of-function mutations (i.e., $\nu_- > \nu_+$), the former is ensured if $s_r < s_p$, that is, if expressing the regulator is less expensive that the cost of the incorrect expression profile of the product, yielding once again $T^* = T_b$.

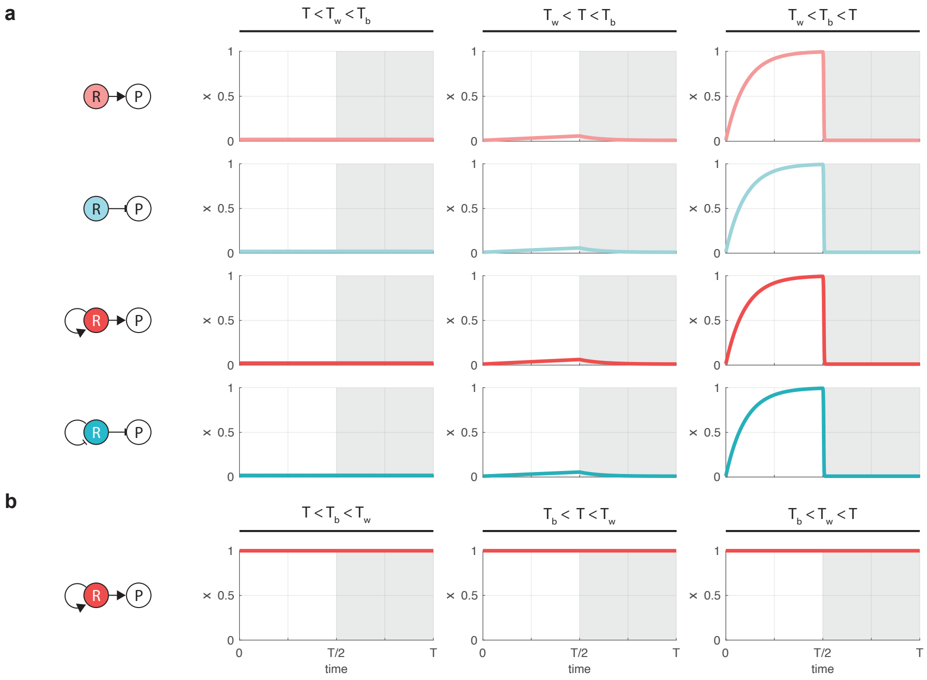

Supplementary Fig. 24. **Fraction of non-binders during each period as a function of the period length.** Red and dark indicates activation and repression, light and dark denotes non-autoregulated and autoregulated control, gray denotes the selection phase. In all plots $\nu_- = 10^{-7}$, $\nu_+ = \nu_-/10$, $s_p = 100\nu_-$, $D = 0.5$. **a** Build-up occurs slower than wipe-out ($T_w < T_b$). Simulation parameters are $s_r = 0.1 s_p$, together with $T = 10^4$, $T = 10^6$, and $T = 10^8$ to yield $T < T_w < T_b$, $T_w < T < T_b$, and $T_w < T_w < T$, respectively, since $T_b = 10^7$ and $T_w = 10^5$. **b** Build-up occurs faster than wipe-out ($T_b < T_w$), which can only happen for positive autoregulation and only if $s_r > s_p + \nu_+ - \nu_-$. Simulation parameters are $s_r = 0.9999 s_p$, together with $T = 10^6$, $T = 10^8$, and $T = 10^{10}$ to yield $T < T_b < T_w$, $T_b < T < T_w$, and $T_b < T_w < T$, respectively, since $T_b = 10^7$ and $T_w = 10^9$.

5 Delay can cause non-autoregulated motifs to outperform autoregulation

In Supplementary Fig. 25, we present simulation data to illustrate how delay affects the performance of autoregulation, and thus the emerging dominant strategies in small populations.

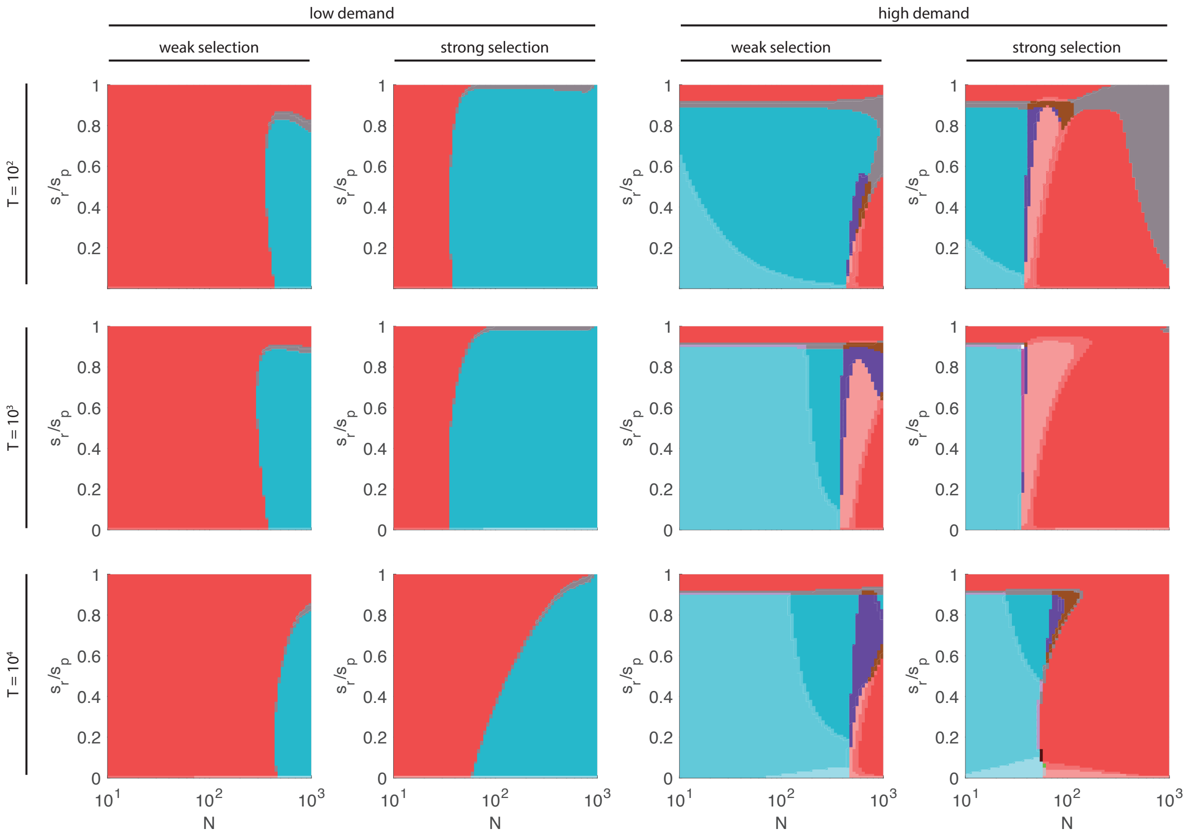

Supplementary Fig. 25. **Evolutionary advantageous regulatory motifs in the presence of delay in small populations.** Data are obtained using the diffusion approximation detailed in the Methods (compare to Supplementary Fig. 21). Colors from Supplementary Table 1 indicate the control scheme(s) with the lowest fitness cost $\bar{s}$: in case of a single winner, the second best is dominated by at least 1%; in case of multiple winners, their difference is less than 1% and all other variants are at least 1% worse. Simulation parameters are: $\nu_- = 10^{-3}$, $\nu_+ = \nu_-/10$. In case of autoregulation, the delay is $t_d = T/100$. Low and high demand refers to $D = 0.05$ and $D = 0.95$, whereas weak and strong selection indicates $s_p = 10\nu_-$ and $s_p = 100\nu_-$, respectively. The period length T is chosen to ensure that $T = 0.1/\nu_-$ in the top row, $T = 1/\nu_-$ in the middle row, and $T = 10/\nu_-$ in the bottom row.

In Supplementary Fig. 26–Supplementary Fig. 27, we present the fraction of non-binders during each period in the presence of delay when considering the deterministic case applicable for large population sizes, together with detailed simulation data in Supplementary Fig. 29–Supplementary Fig. 30 to complement and illuminate the results in Fig. 6. Finally, in Supplementary Fig. 31 we illustrate that the dominant strategy coherent with the use-it-or-lose-it principle does emerge even in the presence of negligible delays, and that greater selection pressure amplifies this effect.

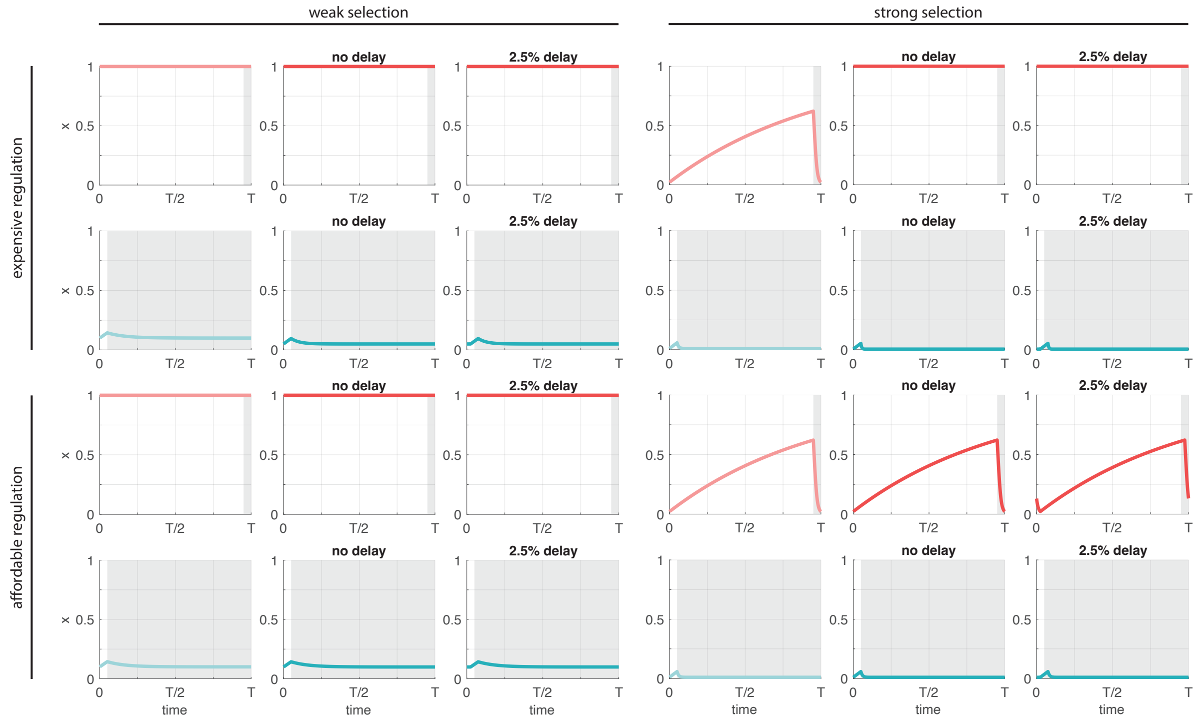

Supplementary Fig. 26. **Fraction of non-binders during each period in the presence of delay (low demand).** Red and blue indicates activation and repression, respectively; light and dark denotes non-autoregulated and autoregulated control, respectively; gray denotes the selection phase. Weak and strong selection indicates $s_p = 10\nu_-$ and $s_p = 100\nu_-$, respectively, whereas affordable and expensive regulation corresponds to $s_r = 0.01 s_p$ and $s_r = 0.99 s_p$, respectively. In all plots $\nu_- = 10^{-7}$, $\nu_+ = \nu_-/10$, $s_p = 10\nu_-$, $D = 0.05$, $T = 10^7$. Delay is indicated as a percentage of the period.

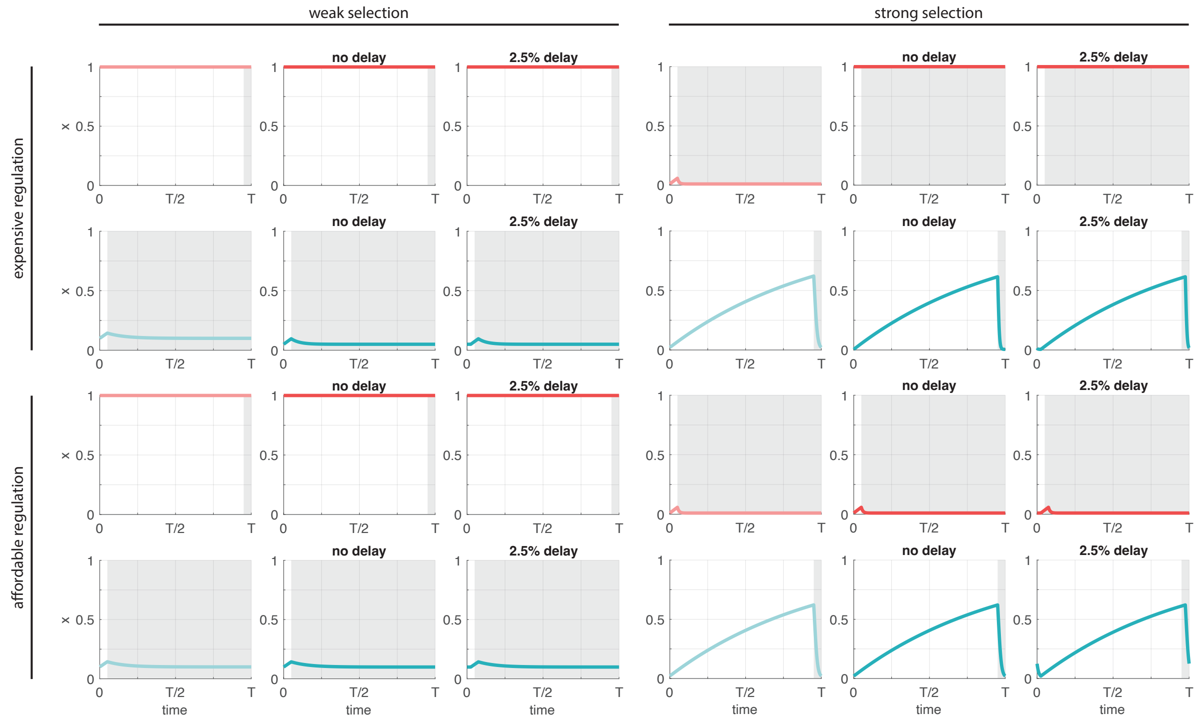

Supplementary Fig. 27. **Fraction of non-binders during each period in the presence of delay (high demand).** Red and blue indicates activation and repression, respectively; light and dark denotes non-autoregulated and autoregulated control, respectively; gray denotes the selection phase. Weak and strong selection indicates $s_p = 10\nu_-$ and $s_p = 100\nu_-$, respectively, whereas affordable and expensive regulation corresponds to $s_r = 0.01s_p$ and $s_r = 0.99s_p$, respectively. In all plots $\nu_- = 10^{-7}$, $\nu_+ = \nu_-/10$, $s_p = 10\nu_-$, $D = 0.95$, $T = 10^7$. Delay is indicated as a percentage of the period.

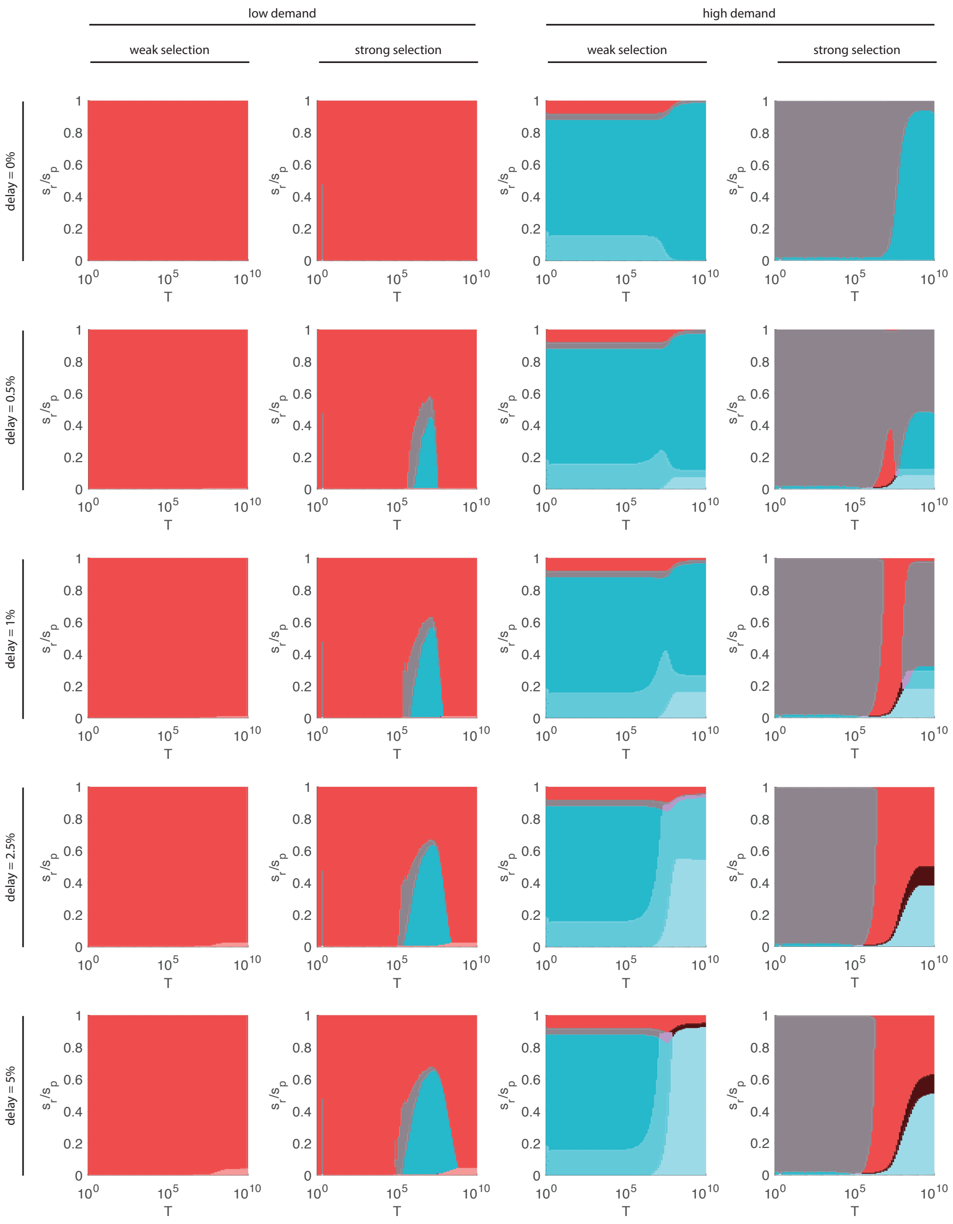

Supplementary Fig. 28. **Evolutionary advantageous regulatory motifs in the presence of delay (low mutation rate).** Colors from Supplementary Table 1 indicate the control scheme(s) with the lowest fitness cost $\bar{s}$: in case of a single winner, the second best is dominated by at least 1%; in case of multiple winners, their difference is less than 1% and all other variants are at least 1% worse. Simulation parameters are: $\nu_- = 10^{-7}$, $\nu_+ = \nu_-/10$. Weak and strong selection indicates $s_p = 10\nu_-$ and $s_p = 100\nu_-$, respectively. Delay is indicated as a percentage of the period.

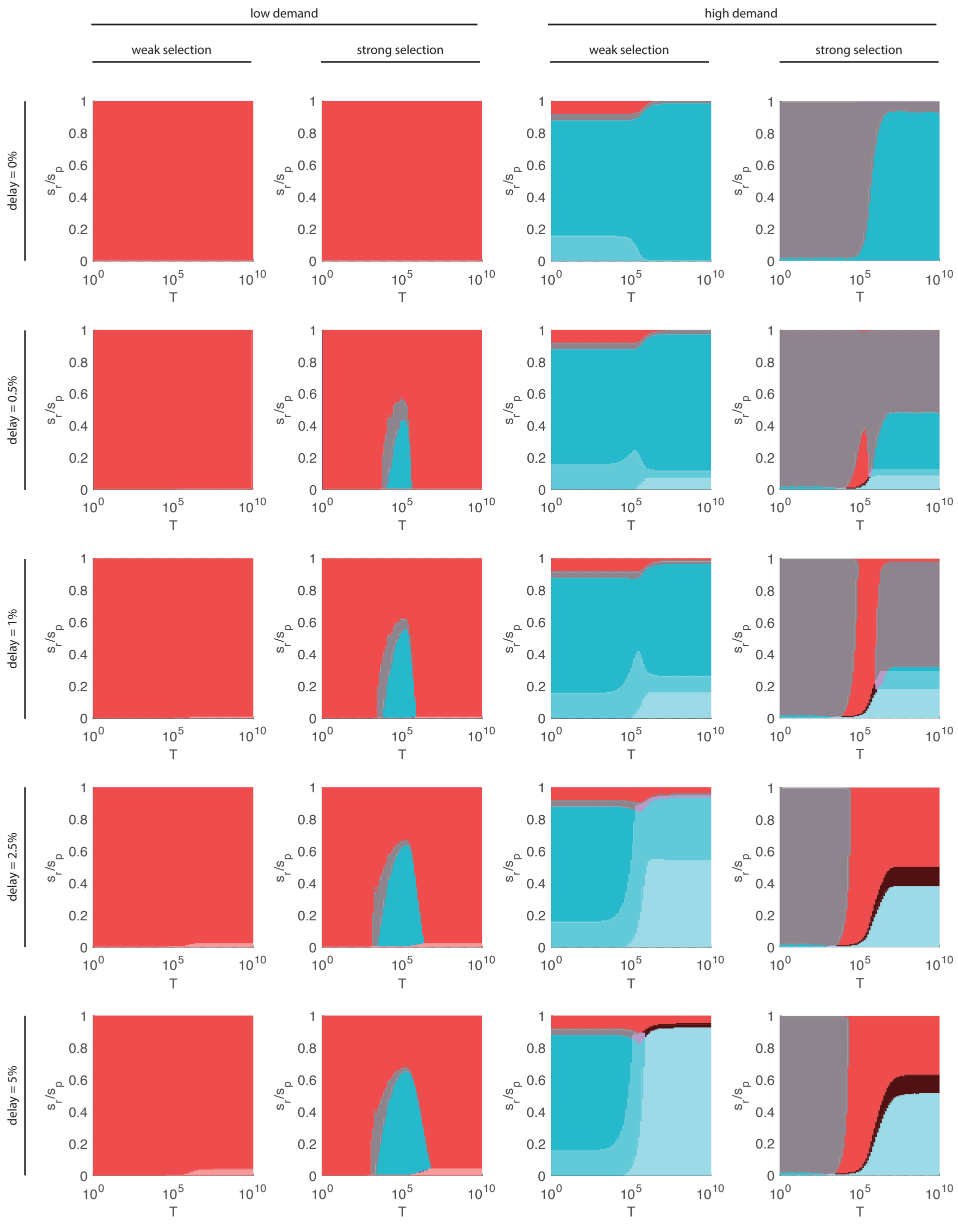

Supplementary Fig. 29. **Evolutionary advantageous regulatory motifs in the presence of delay (medium mutation rate).** Colors from Supplementary Table 1 indicate the control scheme(s) with the lowest fitness cost $\bar{s}$: in case of a single winner, the second best is dominated by at least 1%; in case of multiple winners, their difference is less than 1% and all other variants are at least 1% worse. Simulation parameters are: $\nu_- = 10^{-5}$, $\nu_+ = \nu_-/10$. Weak and strong selection indicates $s_p = 10\nu_-$ and $s_p = 100\nu_-$, respectively. Delay is indicated as a percentage of the period.

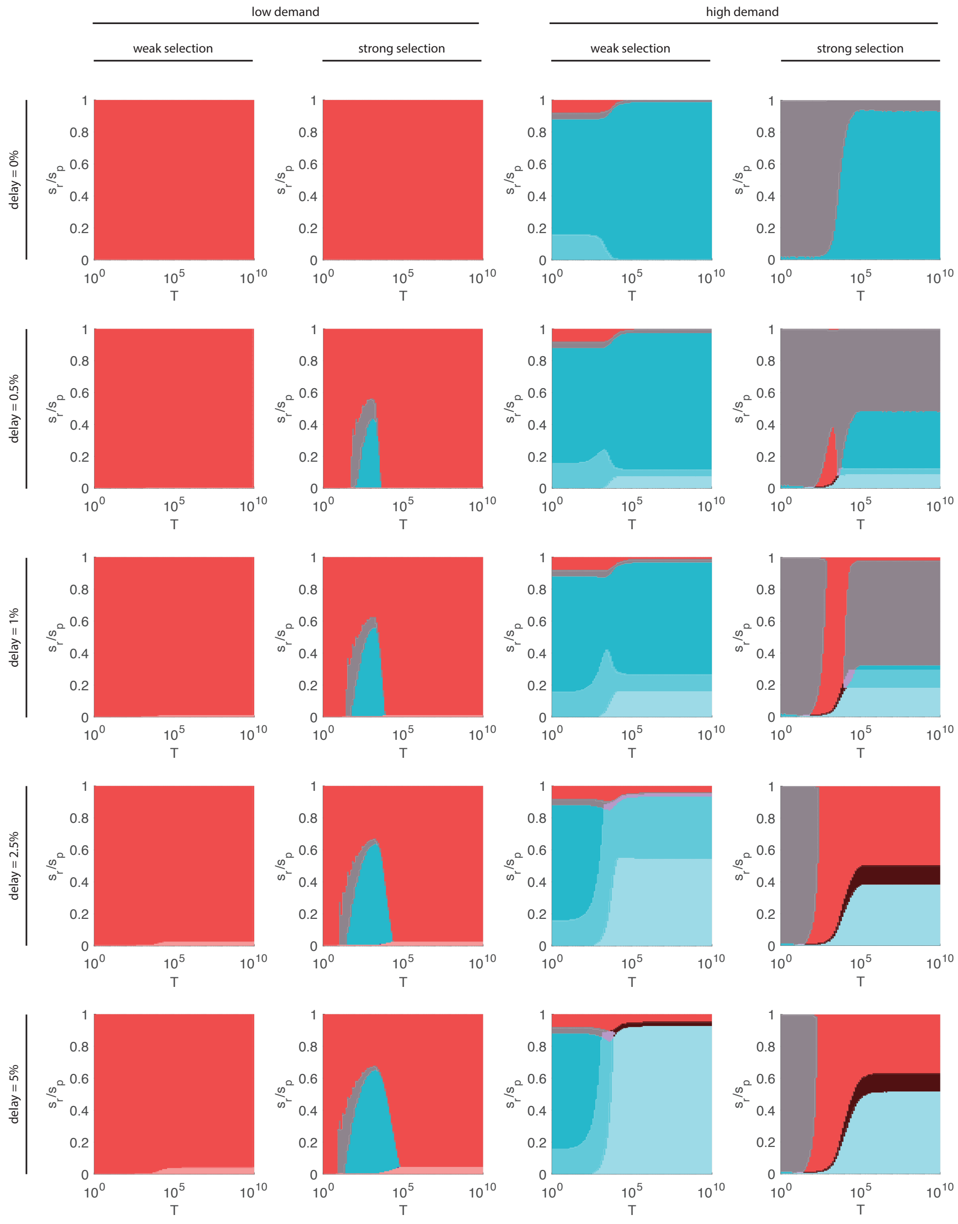

Supplementary Fig. 30. **Evolutionary advantageous regulatory motifs in the presence of delay (high mutation rate).** Colors from Supplementary Table 1 indicate the control scheme(s) with the lowest fitness cost $\bar{s}$: in case of a single winner, the second best is dominated by at least 1%; in case of multiple winners, their difference is less than 1% and all other variants are at least 1% worse. Simulation parameters are: $\nu_- = 10^{-3}$, $\nu_+ = \nu_-/10$. Weak and strong selection indicates $s_p = 10\nu_-$ and $s_p = 100\nu_-$, respectively. Delay is indicated as a percentage of the period.

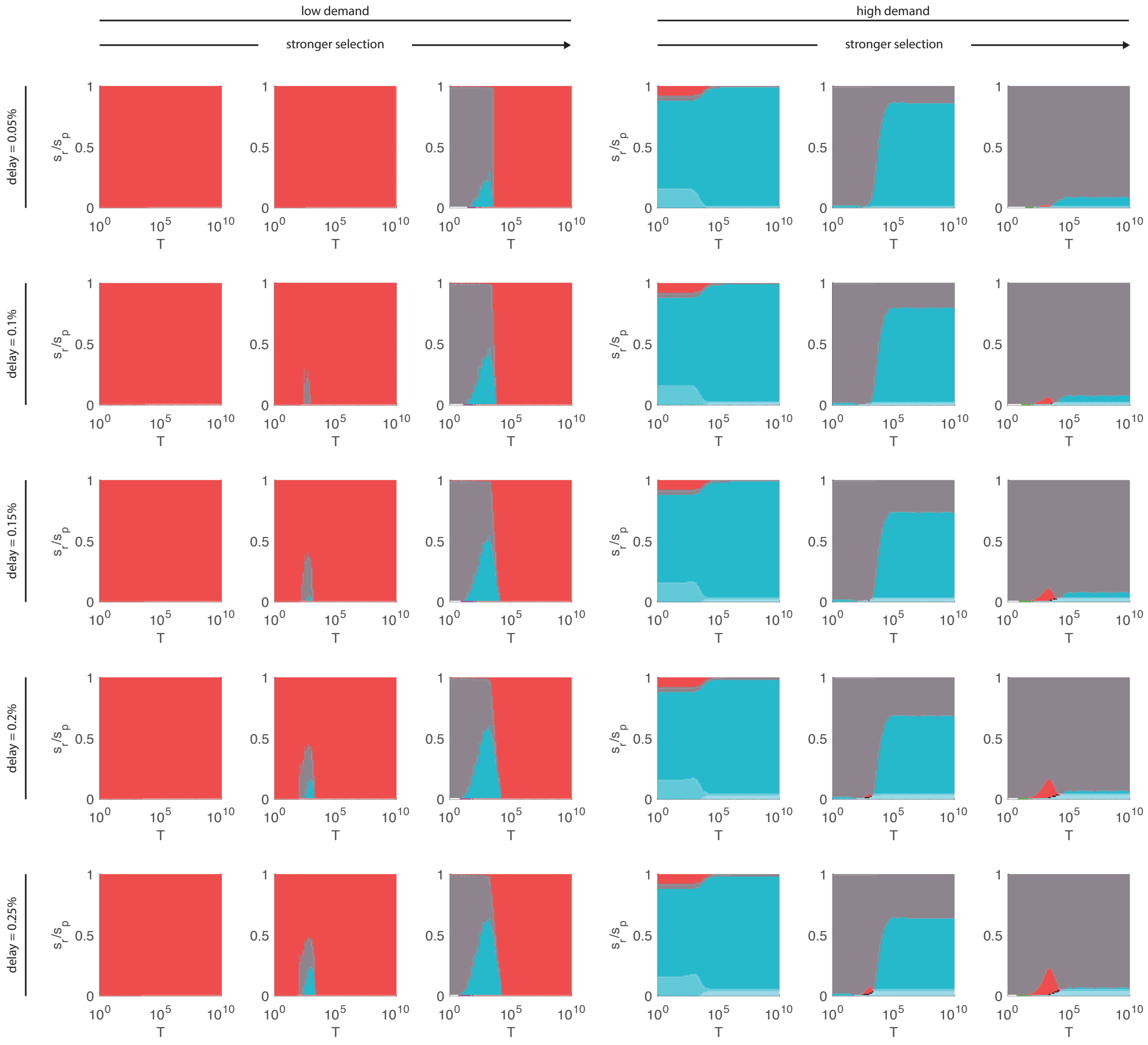

Supplementary Fig. 31. **Even negligible delay can result in the emergence of the dominant strategy that is coherent with the use-it-or-lose-it principle, and greater selection pressure amplifies this effect.** Colors from Supplementary Table 1 indicate the control scheme(s) with the lowest fitness cost $\bar{s}$: in case of a single winner, the second best is dominated by at least 1%; in case of multiple winners, their difference is less than 1% and all other variants are at least 1% worse. Simulation parameters are: $\nu_- = 10^{-3}$, $\nu_+ = \nu_-/10$. Selection strength increases from $s_p = 10\nu_-$ through $s_p = 100\nu_-$ to $s_p = 1000\nu_-$. Low and high demand refers to $D = 0.05$ and $D = 0.95$, respectively. Delay is indicated as a percentage of the period.

6 Feedback cost can cause non-autoregulated motifs to outperform autoregulation

In Supplementary Fig. 32–Supplementary Fig. 34 we include detailed simulation data complementing the results presented in Fig. 7 to illustrate that (i) the pattern of dominant strategies remains largely unaffected from the perspective of wear-and-tear or use-it-or-lose-it principle; (ii) non-autoregulated strategies can outperform their autoregulated counterparts as a increases; and (iii) this transition happens at lower values of a for repression than for activation.

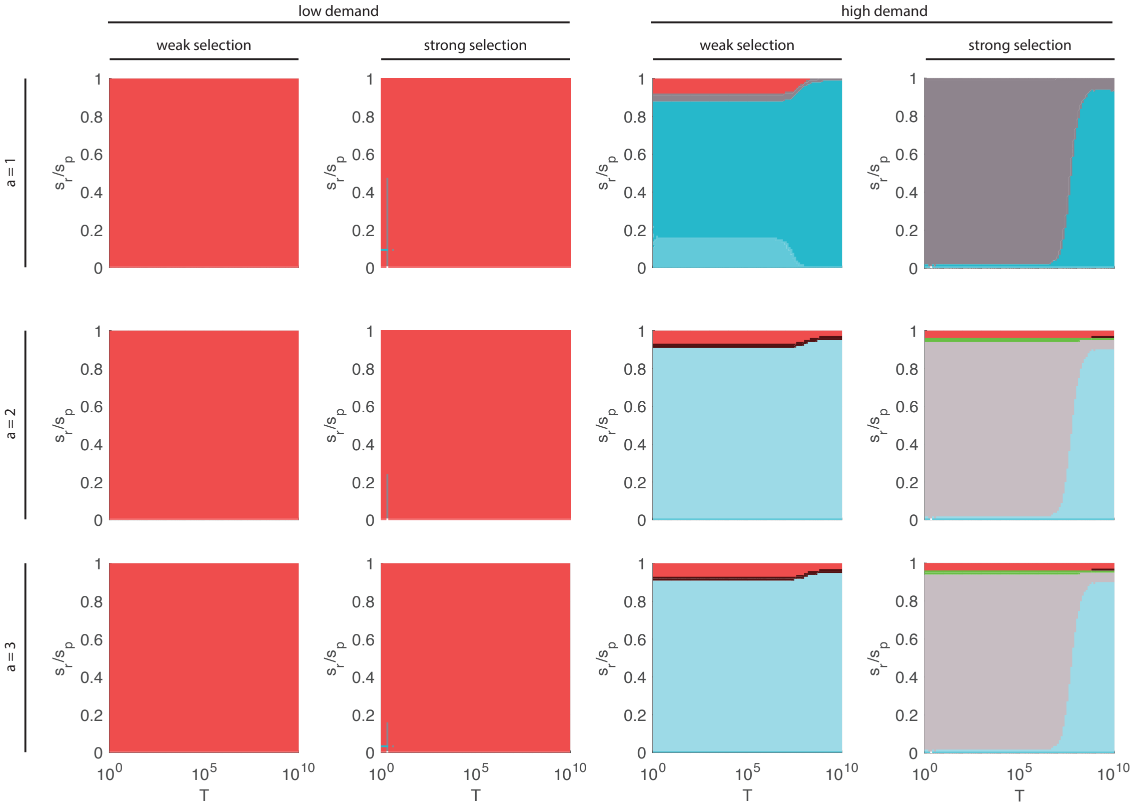

Supplementary Fig. 32. **Evolutionary advantageous regulatory motifs in the presence of additional fitness cost due to autoregulation (low mutation rate).** Colors from Supplementary Table 1 indicate the control scheme(s) with the lowest fitness cost $\bar{s}$: in case of a single winner, the second best is dominated by at least 1%; in case of multiple winners, their difference is less than 1% and all other variants are at least 1% worse. Simulation parameters are: $\nu_- = 10^{-7}$, $\nu_+ = \nu_-/10$. Weak and strong selection indicates $s_p = 10\nu_-$ and $s_p = 100\nu_-$, respectively. Low and high demand refers to $D = 0.05$ and $D = 0.95$, respectively.

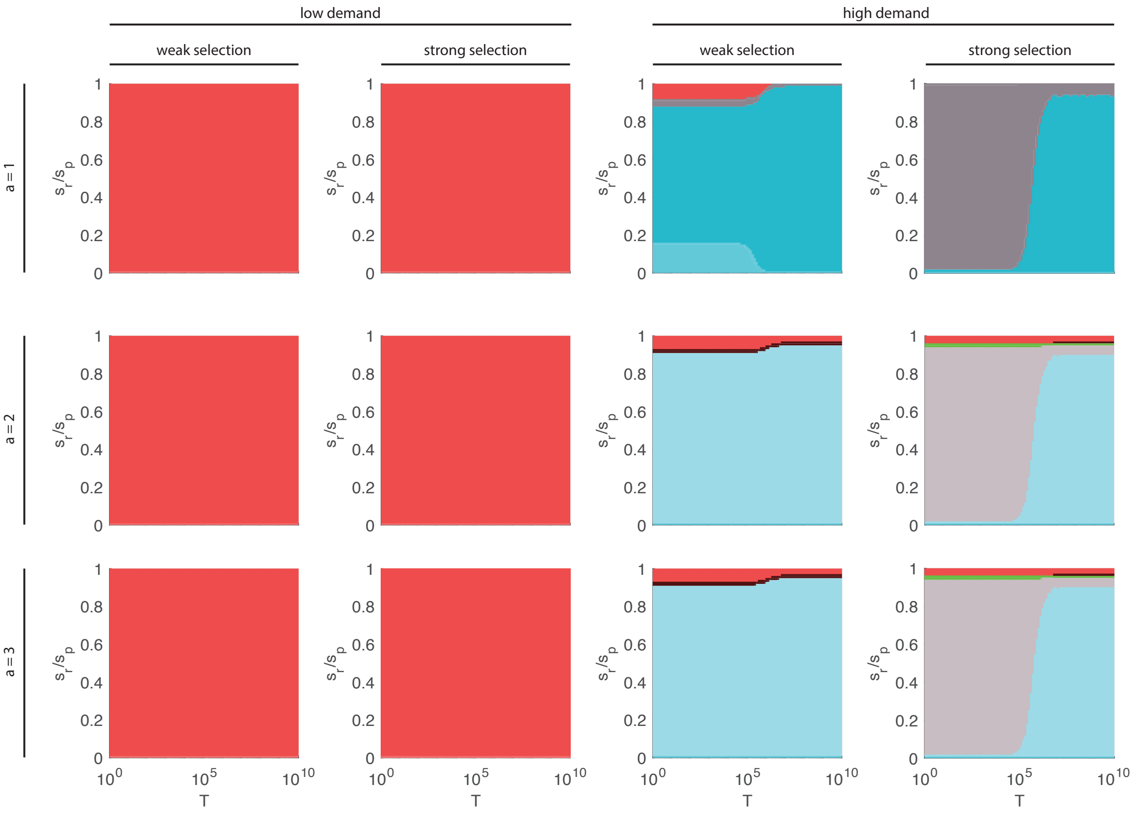

Supplementary Fig. 33. **Evolutionary advantageous regulatory motifs in the presence of additional fitness cost due to autoregulation (medium mutation rate).** Colors from Supplementary Table 1 indicate the control scheme(s) with the lowest fitness cost $\bar{s}$: in case of a single winner, the second best is dominated by at least 1%; in case of multiple winners, their difference is less than 1% and all other variants are at least 1% worse. Simulation parameters are: $\nu_- = 10^{-5}$, $\nu_+ = \nu_-/10$. Weak and strong selection indicates $s_p = 10\nu_-$ and $s_p = 100\nu_-$, respectively. Low and high demand refers to $D = 0.05$ and $D = 0.95$, respectively.

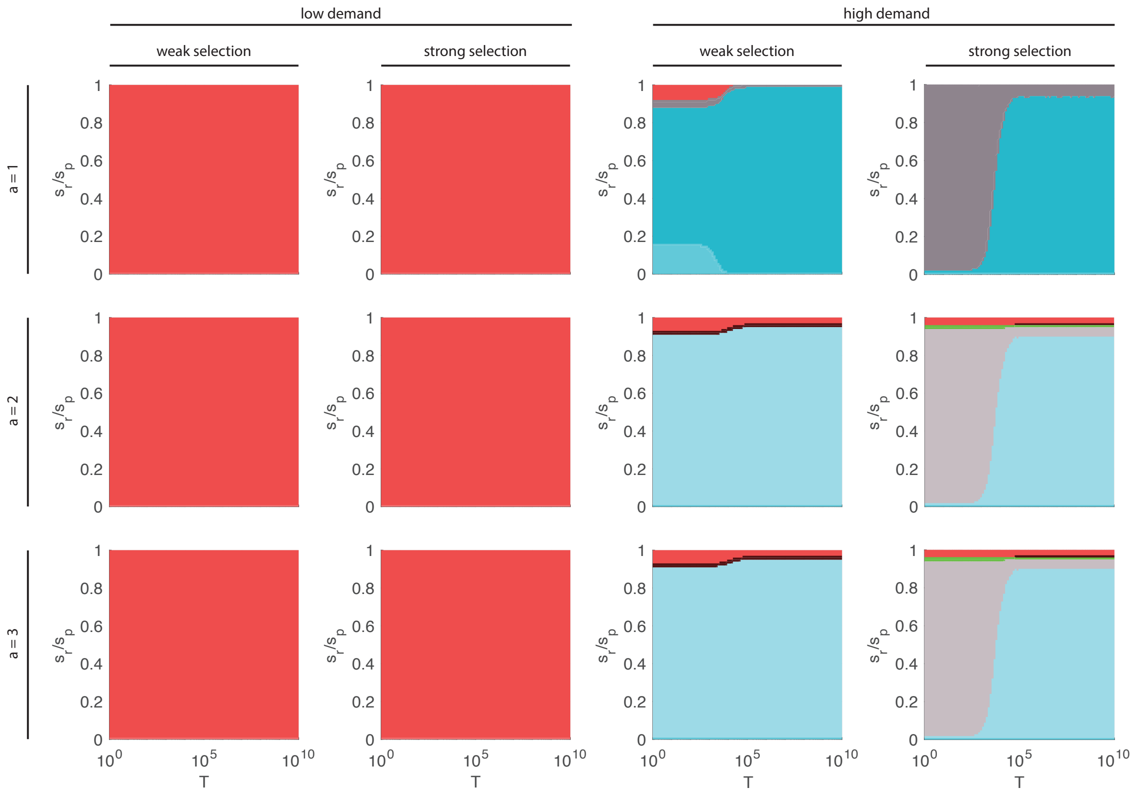

Supplementary Fig. 34. **Evolutionary advantageous regulatory motifs in the presence of additional fitness cost due to autoregulation (high mutation rate).** Colors from Supplementary Table 1 indicate the control scheme(s) with the lowest fitness cost $\bar{s}$: in case of a single winner, the second best is dominated by at least 1%; in case of multiple winners, their difference is less than 1% and all other variants are at least 1% worse. Simulation parameters are: $\nu_- = 10^{-3}$, $\nu_+ = \nu_-/10$. Weak and strong selection indicates $s_p = 10\nu_-$ and $s_p = 100\nu_-$, respectively. Low and high demand refers to $D = 0.05$ and $D = 0.95$, respectively.

7 Reduced bioenergetic cost may contribute to the prevalence of autoregulation in model organisms

While CoryneRegNet 7 primarily focuses on corynebacterial regulatory interactions, it also offers a convenient platform to access data about other organisms (*2*), containing up-to-date regulatory information about *Corynebacterium glutamicum ATCC 13032* (*C. glutamicum*) based on CoryneRegNet 6 (*3*) updated with new data (*4*), about *Escherichia coli K-12* (*E. coli*) based on RegulonDB (*5*), about *Bacillus subtilis* (*B. subtilis*) based on DBTBS (*6*), and about *Mycobacterium tuberculosis H37Rv* (*M. tuberculosis*) based on (*7*). While (*7*) reveals thousands of protein-DNA binding events, it does not integrate all known interactions: e.g., no TF is reported to be negatively autoregulated, despite experimentally documented cases of self-repression (*8, 9*). Conversely, on average 42% of TFs are self-repressed (SD: 6%) considering *E. coli*, *B. subtilis*, and *C. glutamicum*, suggesting that the dataset for *M. tuberculosis* may be less comprehensive. Therefore, we decided not to include *M. tuberculosis* in our analysis.

For other organisms, data availability is considerably more limited (Supplementary Fig. 35). In particular, for those featured in the state-of-the-art PRODORIC database (*10*) that contains curated prokaryotic gene regulation data for almost 30 organisms, the average number of TFs is 7 (SD: 8). Conversely, for *E. coli*, *B. subtilis*, and *C. glutamicum* from (*2*) the average is 136

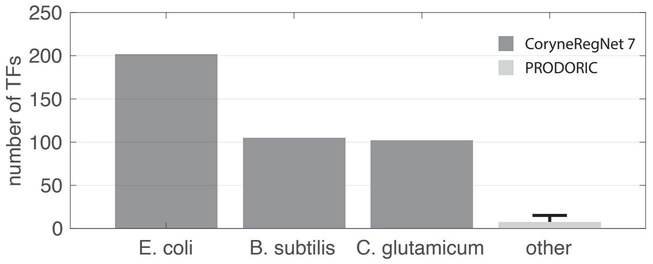

Supplementary Fig. 35. **Data availability about transcriptional regulatory networks.** The number of TFs contained in CoryneRegNet 7 (*2*) for *E. coli*, *B. subtilis*, and *C. glutamicum* is considerably greater than for other organisms in the PRODORIC database (*10*).

Supplementary Table 2. **Statistical data for the analysis in Fig. 8.** Number of trials is denoted by n_r and n, the number of successes are given by X_r and X in the reference and in the sample of interest, respectively (see Methods). The labels "baseline" and "modified baseline" refer to the baselines used in Fig. 8ac and Fig. 8d, respectively, whereas "sample of interest" indicates synergistic regulators.

		baseline		modified baseline		sample of interest	
		n_r	X_r	n_r	X_r	n	X
B. subtilis	self-repression	105	36	466	167	224	118
	self-activation	105	8	466	74	117	41
C. glutamicum	self-repression	102	46	1460	699	476	314
	self-activation	102	8	1460	325	247	123
E. coli	self-repression	202	92	4453	2729	626	421
	self-activation	202	38	4453	1119	777	303

(SD: 57). Therefore, data for Fig. 8 were downloaded from `https://exbio.wzw.tum.de/coryneregnet/`, for *B. subtilis*, *C. glutamicum*, and *E. coli*. As some TFs may act as both activators and repressors depending on cellular conditions (*11, 12*), interactions that were indicated as both positive and negative were considered as both. Interactions that were recorded neither positive, negative, or both, were discarded. In Supplementary Table 2 we include the data underpinning the statistical analysis in Fig. 8.

We next show that if reduced R-cost contributes to the preferential selection of autoregulated TFs (in addition to other beneficial properties), then their prevalence among all regulators should be exceeded by their frequency in our samples of interest. While our samples of interest comprise TFs controlling the expression of synergistically regulated target genes, the synthesis of other genes may be under the antagonistic control of TFs. Thus, in Supplementary Fig. 36 we focus on genes that are regulated by two TFs either synergistically or antagonistically (Fig. 8b). The variable $q \in [0, 1]$ captures how loss-of-function mutations affecting only one of the regulators impact the P-cost. In what follows, we assume that $q = 0.5$, corresponding to the case when the impact on the P-cost from the required non-binder regulators is identical and additive. Other scenarios can be analyzed similarly by choosing a different value of q.

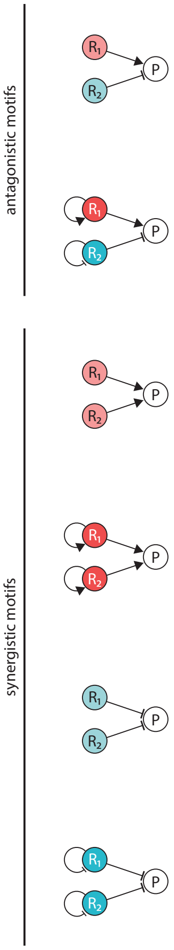

		induced		non-induced	
		R_2		R_2	
		binder	non-binder	binder	non-binder
R_1	binder	$2s_r$	$2s_r$	$2s_r$	$2s_r+s_p$
	non-binder	$2s_r+s_p$	$2s_r+s_p$	$2s_r$	$2s_r+s_p$

		induced		non-induced	
		R_2		R_2	
		binder	non-binder	binder	non-binder
R_1	binder	$2s_r$	$2s_r$	0	s_r+s_p
	non-binder	s_r+s_p	s_r+s_p	0	s_r+s_p

		induced		non-induced	
		R_2		R_2	
		binder	non-binder	binder	non-binder
R_1	binder	$2s_r$	$2s_r+qs_p$	$2s_r$	$2s_r$
	non-binder	$2s_r+qs_p$	$2s_r+s_p$	$2s_r$	$2s_r$

		induced		non-induced	
		R_2		R_2	
		binder	non-binder	binder	non-binder
R_1	binder	$2s_r$	s_r+qs_p	0	0
	non-binder	s_r+qs_p	s_p	0	0

		induced		non-induced	
		R_2		R_2	
		binder	non-binder	binder	non-binder
R_1	binder	$2s_r$	$2s_r$	$2s_r$	$2s_r+qs_p$
	non-binder	$2s_r$	$2s_r$	$2s_r+qs_p$	$2s_r+s_p$

		induced		non-induced	
		R_2		R_2	
		binder	non-binder	binder	non-binder
R_1	binder	$2s_r$	$2s_r$	0	s_r+qs_p
	non-binder	$2s_r$	$2s_r$	s_r+qs_p	$2s_r+s_p$

Supplementary Fig. 36. **Fitness cost of core regulatory motifs with two regulators.** Deleterious loss-of-function mutations affecting either of the regulators may result in the incorrect expression profile of the product during the selection phase, captured via $q \in [0, 1]$. For antagonistic motifs, q does not appear as the activator and repressor behave as if they were functional during the non-induced and induced phases, respectively, even if they suffer from loss-of-function mutations.

Let $x_{b,nb}(t)$, $x_{nb,b}(t)$, $x_{nb,nb}(t)$, and $x_{b,b}(t)$ denote the fraction of the population equipped with binder R_1 and non-binder R_2, non-binder R_1 and binder R_2, non-binder R_1 and R_2, and binder R_1 and R_2, respectively. From Supplementary Fig. 36, let $s_{b,nb}(t)$, $s_{nb,b}(t)$, $s_{nb,nb}(t)$, and $s_{b,b}(t)$ denote the corresponding fitness costs, respectively. Generalizing the expression in (11),

the population's average fitness cost during each period can be calculated as

$$\bar{s} = \frac{1}{T}\int_0^T \left[x_{nb,nb}(t)s_{nb,nb}(t) + x_{b,nb}(t)s_{b,nb}(t) + x_{nb,b}(t)s_{nb,b}(t) + x_{b,b}(t)s_{b,b}(t)\right] \mathrm{d}t. \qquad (12)$$

Considering the typical range of mutation rates and timescale of environmental shifts, we computed the average fitness cost of all motifs featured in Supplementary Fig. 36 for varying levels of the demand and selection pressure (Supplementary Fig. 37). Notably, only the two synergistic autoregulated motifs emerged as dominant. This highlights not only that autoregulation of TFs provides an evolutionary advantage (echoing the findings featured in Fig. 3), but also that synergistic regulation outperforms antagonistic motifs in case of multiple regulators.

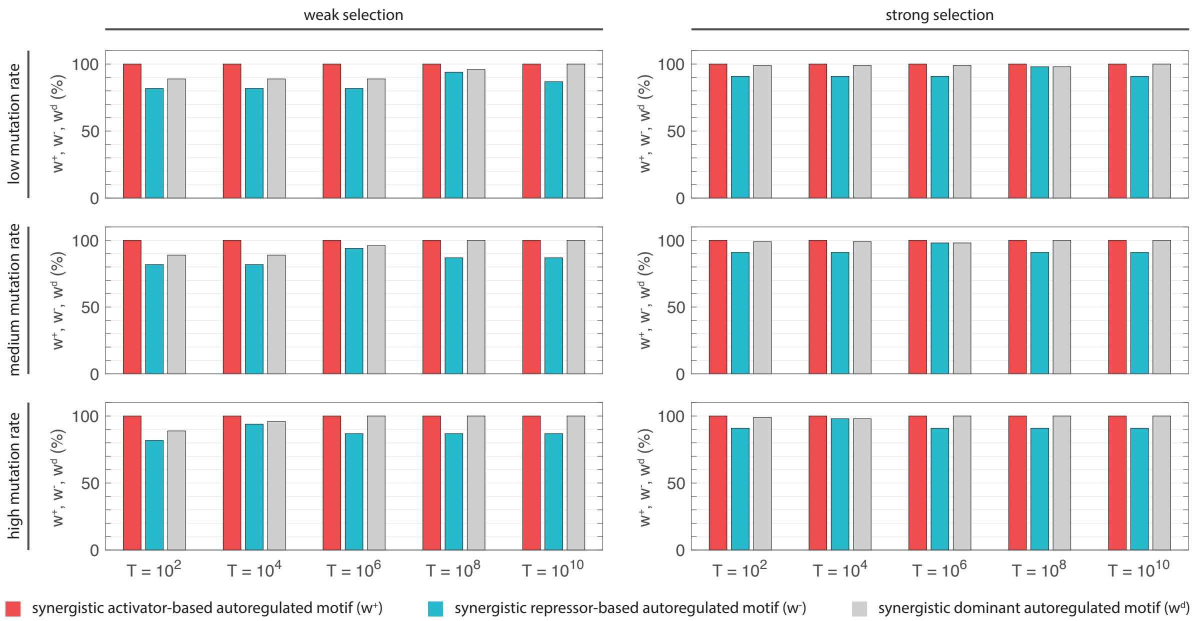

Supplementary Fig. 37. **Relative fitness cost improvement upon autoregulation.** In all plots, simulation parameters are $\nu_+ = \nu_-/10$, $s_r = 0.1s_p$. Weak and strong selection refer to $s_p = 10\nu_-$ and $s_p = 100\nu_-$, respectively. Low, medium, and high mutation rate corresponds to $\nu_- = 10^{-7}$, $\nu_- = 10^{-5}$, and $\nu_- = 10^{-3}$, respectively. The average fitness cost $\bar{s}$ is computed according to (12) for all motifs featured in Supplementary Fig. 36 considering varying levels of the demand ($D = 0.01, 0.02, \ldots, 0.99$). Red and blue bars represent the percentage of cases (across the values of D) when $\Delta s^+ < 1$ and $\Delta s^- < 1$, respectively. Gray bars indicate the percentage of cases (across the values of D) when relative fitness cost improvement (either Δs^+ or Δs^-) corresponding to the dominant motif is smaller than one.

Next, we focused on the relative fitness cost decrease when equipping the non-autoregulated motifs with autoregulation. To this end, introduce s^{++} and s^{++}_{auto} for the average fitness cost of the positive synergistic motif without and with autoregulation, respectively, and define s^{--}, s^{--}_{auto} for the negative synergistic motif, and s^{+-}, s^{+-}_{auto} for the antagonistic motif similarly. As a result,

$$\delta s^{++} = \frac{s^{++}_{auto}}{s^{++}}, \qquad \delta s^{--} = \frac{s^{--}_{auto}}{s^{--}}, \qquad \delta s^{+-} = \frac{s^{+-}_{auto}}{s^{+-}}$$

quantify the relative fitness cost change when equipping the non-autoregulated motifs with autoregulation. Similarly, introduce $\Delta s^{+} = \delta s^{++}/\delta s^{+-}$ and $\Delta s^{-} = \delta s^{--}/\delta s^{+-}$, so that if $\Delta s^{+} < 1$ then the relative fitness gain is greater for the positive synergistic motif than for the antagonistic one, and similarly for the negative synergistic motif if $\Delta s^{-} < 1$. In other words, the selection pressure to equip the regulators with autoregulation is greater for the corresponding synergistic motifs than for their antagonistic counterpart.

Data in Supplementary Fig. 37 suggest that synergistic motifs generally enjoy a greater relative fitness cost improvement upon autoregulation across a broad range of typical parameter combinations. In particular, $\Delta s^{+} < 1$ for $w^{+} = 100\%$ of the cases (red in Supplementary Fig. 37), whereas $\Delta s^{-} < 1$ in $w^{-} = 89\%$ of them (blue in Supplementary Fig. 37). Regarding the latter, $\Delta s^{-} > 1$ typically occurs when the positive synergistic autoregulated motif is the dominant instead of the negative variant (e.g., in the low demand case as the dominant motif is typically underpinned by the wear-and-tear principle). When considering the motif that emerges as dominant, the corresponding relative fitness cost improvement (either Δs^{+} or Δs^{-}) is smaller than one in $w^{d} = 97\%$ of the cases (gray in Supplementary Fig. 37). Taken together, these results suggest that we should expect stronger pressure to augment synergistic non-autoregulated motifs with autoregulation than their antagonistic counterpart if reduced R-cost was a contributing factor, in accordance with the data presented in Fig. 8 (though other factors may also play a role in explaining the observed overrepresentation).

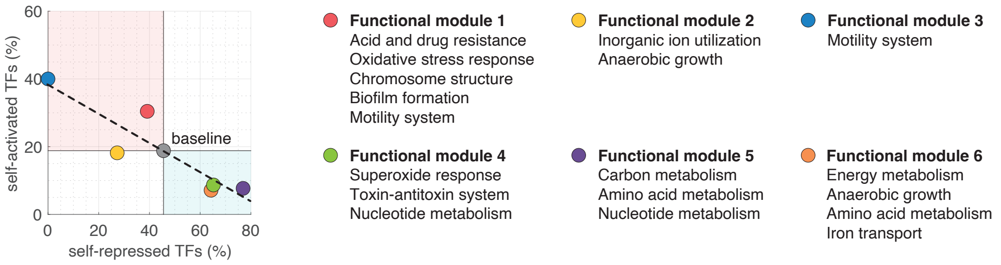

Supplementary Fig. 38. **Prevalence of autoregulation in functional modules of *E. coli*.** Functional modules are identified in (*13*) using transcriptomics data (*14*). The gray circle denotes the baseline prevalence of autoregulation among all TFs. The dashed line illustrates the result of linear regression (slope: -0.43, offset: 38.25) considering the prevalence of autoregulation among the six functional modules (but not the baseline).

Finally, we focused on the functional modules that were identified in *E. coli* (*13*) based on transcriptomics data (*14*). Considering the prevalence of autoregulation in these modules that represent core biological functions, there is a negative correlation between the frequency of self-activation and self-repression (dashed line in Supplementary Fig. 38). Comparing the prevalence of these motifs to the baseline observed across all TFs in the dataset (self-repression: 63.9%, self-activation: 25.4%, gray circle in Supplementary Fig. 38), three of the modules appear to be dominated by self-activation (red region in Supplementary Fig. 38) and three by self-repression (blue region in Supplementary Fig. 38). This could serve as a promising starting point for further investigating the potential role that the reduced bioenergetic cost of autoregulation may play in contributing to its prevalence. For instance, functional module 3 encapsulates the motility system, and comprises five TFs of which two are self-activated (LrhA and RcsA). At the other end of the spectrum, functional module 5 encompasses the metabolism of carbon, amino acids, and nucleotides, and comprises 13 TFs of which ten are self-repressed (ArgR, CysB, CytR, GalR, GalS, LsrR, MelR, PepA, PurR, and TrpR). Focusing on the demand profile of these TFs could thus further clarify the potential relationship between the demand for a beneficial gene product and the evolutionary advantage that autoregulation may confer.

8 Autoregulation can impact genetic stability in synthetic biology applications

In Supplementary Fig. 39 we consider a collection of population sizes typical in synthetic biology applications, spanning the range from microfluidics-based to bioreactor-based experimental contexts (*15–19*). Instead of an evolutionary timescale, we assume that environmental shifts occur much faster with a period of $T = 10$ generations, and we are interested in how rapidly non-binders emerge after only $T_f = 100$ generations. For instance, in *E. coli* growing under laboratory conditions, these correspond to approximately 5 hours and 2 days based on typical doubling times (*20*). The results reveal two key observations across population sizes.

First, focusing only on non-autoregulated control, positive and negative regulation offer practically identical performance considering the emergence of non-binders for a given selection duration and strength (which follows from Fig. 2c). Therefore, non-autoregulated activation in the low/high demand case and non-autoregulated repression in the high/low demand case are

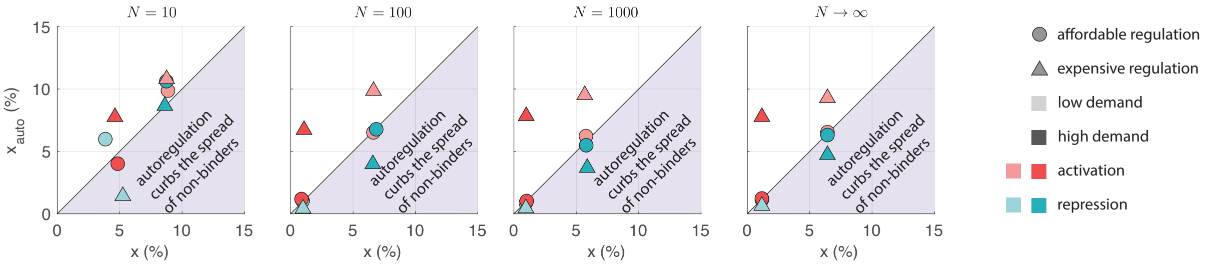

Supplementary Fig. 39. **Autoregulation can considerably impact the emergence and spread of non-binders in synthetic biology applications.** For $N = 10$, $N = 100$, and $N = 1000$, data were obtained using the stochastic sampling algorithm described in the Methods for 1000 independent simulations. For $N \to \infty$ the deterministic dynamics in (7) were simulated. Non-binders are absent at the beginning of each simulation. The fraction of non-binders are measured at $T_f = 100$, x and x_{auto} denotes its value in case of non-autoregulated and autoregulated motifs, respectively. In all plots $\nu_- = 10^{-3}$, $\nu_+ = \nu_-/10$, $s_p = 100\nu_-$, $T = 10$. Red and blue denotes activation and repression, respectively. Light and dark shades indicate low demand ($D = 0.1$) and high demand ($D = 0.9$), respectively. Circles and triangles correspond to affordable ($s_r = 0.1 s_p$) and expensive regulation ($s_r = 0.9 s_p$), respectively.

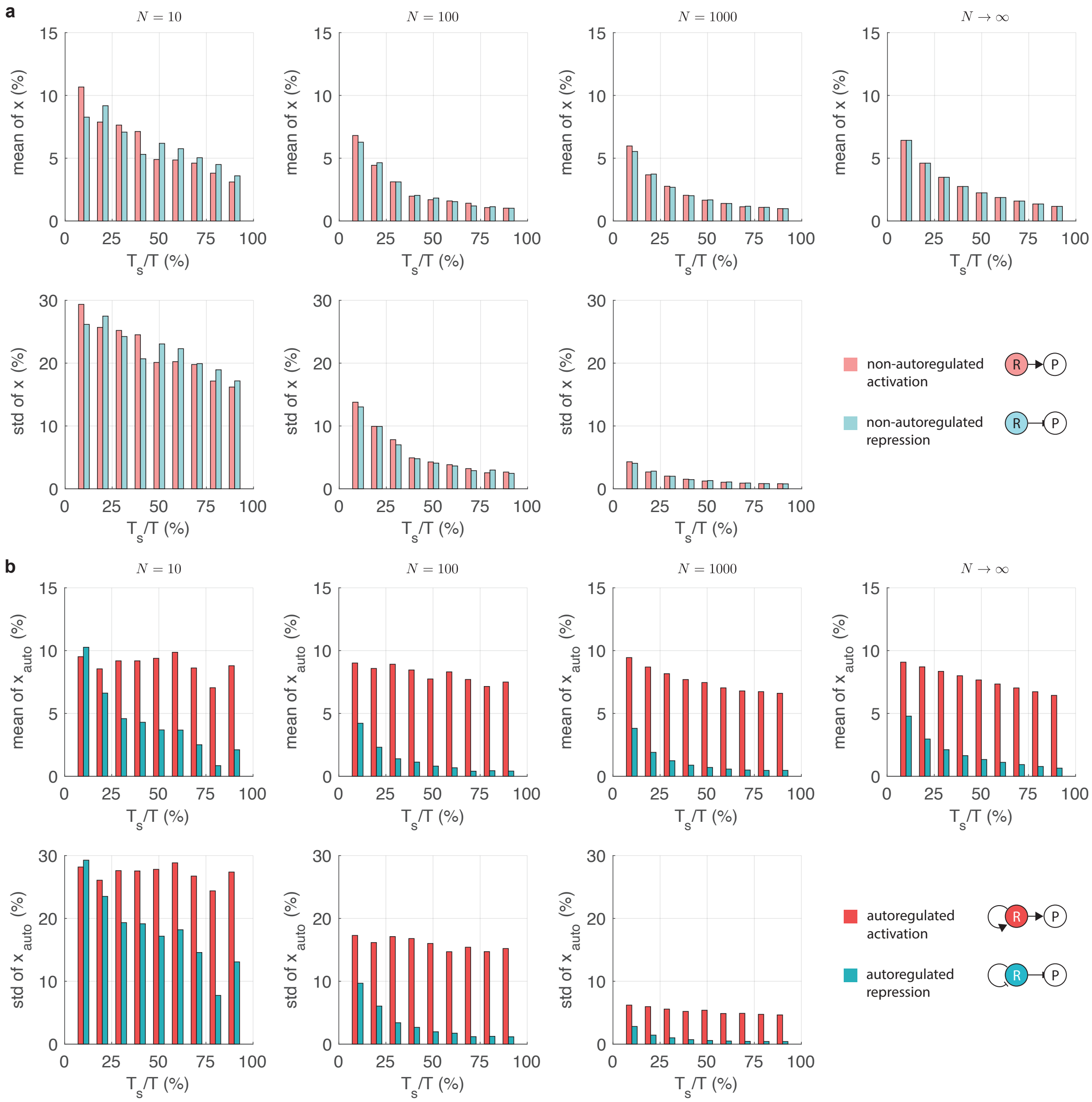

Supplementary Fig. 40. **Population level composition and spread of non-binders for typical population sizes in synthetic biology applications.** For $N < \infty$ data were obtained using the stochastic sampling algorithm described in the Methods for 1000 independent simulations. For $N \to \infty$ the deterministic dynamics in (7) were simulated. Non-binders are absent at the beginning of each simulation. The fraction of non-binders x is measured at $T_f = 100$. In all plots $\nu_- = 10^{-3}$, $\nu_+ = \nu_-/10$, $s_p = 100\nu_-$, $T = 10$, $s_r = 0.9s_p$. The standard deviation for the deterministic case is not displayed as it is zero. **a** Mean and standard deviation of x for non-autoregulated motifs. **b** Mean and standard deviation of x for autoregulated motifs.

interchangeable (Supplementary Supplementary Fig. 40a) as a result of their time-invariant R-cost. This is not true for autoregulation (Supplementary Supplementary Fig. 40b), since their R-cost varies over time. These results hold across a wide range of population sizes typical in synthetic biology applications.

Second, the difference between non-autoregulated and autoregulated control increases with the cost of regulation. When regulation is affordable (circles in Supplementary Fig. 39), the two modes encounter similar fitness cost as the reduction in R-cost plays a minor role (Fig. 2c). Conversely, as the fitness cost gain of autoregulation increases with the price of regulation by eliminating the gratuitous expression of the regulator (Fig. 2c), non-autoregulated and autoregulated control offer considerably different performance once regulation becomes expensive (triangles in Supplementary Fig. 39). In particular, while self-repression eliminates non-binders,

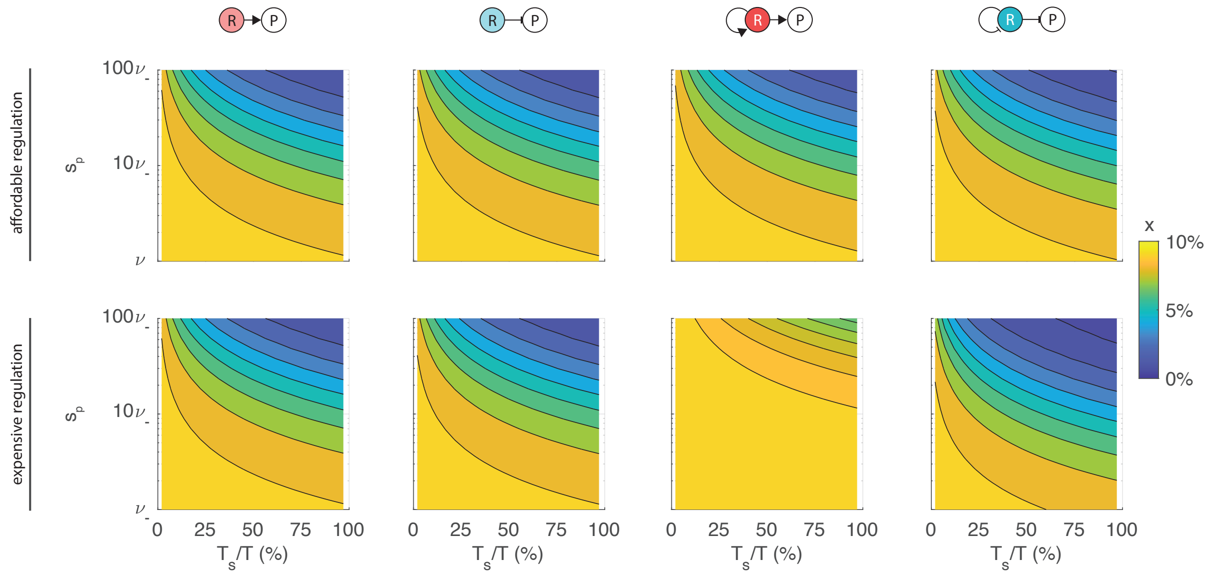

Supplementary Fig. 41. **Fraction of non-binders decreases as selection becomes stronger and longer.** Data were obtained using the deterministic dynamics in (7). Non-binders are absent at the beginning of each simulation. The fraction of non-binders x is measured at $T_f = 100$. In all plots $\nu_- = 10^{-3}$, $\nu_+ = \nu_-/10$, and $T = 10$. Affordable and expensive regulation corresponds to $s_r = 0.1 s_p$ and $s_r = 0.9 s_p$, respectively.

self-activation instead facilitates their spread (Fig. 5). For instance, the former can result in a 25% decrease (dark blue triangle in Supplementary Fig. 39), whereas the latter in a seven-fold increase (dark red triangle in Supplementary Fig. 39) in large populations. Finally, in Supplementary Fig. 41 we confirm that non-binders are eliminated as selection becomes stronger and longer, and while self-repression further enhances this effect, self-activation instead attenuates it (as expected, the impact increases with s_r, and there is no change for non-autoregulated control).

References

1. Gerland, U. & Hwa, T. Evolutionary selection between alternative modes of gene regulation. *PNAS* **106**, 8841–8846 (2009).

2. Parise, M. T. D. *et al.* CoryneRegNet 7, the reference database and analysis platform for corynebacterial gene regulatory networks. *Scientific Data* **7**, 142 (2020).

3. Pauling, J., Rottger, R., Tauch, A., Azevedo, V. & Baumbach, J. CoryneRegNet 6.0–Updated database content, new analysis methods and novel features focusing on community demands. *Nucleic Acids Research* **40**, D610–D614 (2012).

4. Freyre-González, J. A. & Tauch, A. Functional architecture and global properties of the Corynebacterium glutamicum regulatory network: Novel insights from a dataset with a high genomic coverage. *Journal of Biotechnology* **257**, 199–210 (2017).

5. Santos-Zavaleta, A. *et al.* RegulonDB v 10.5: tackling challenges to unify classic and high throughput knowledge of gene regulation in E. coli K-12. *Nucleic Acids Research* **47**, D212–D220 (2018).

6. Sierro, N., Makita, Y., de Hoon, M. & Nakai, K. DBTBS: a database of transcriptional regulation in Bacillus subtilis containing upstream intergenic conservation information. *Nucleic Acids Research* **36**, D93–D96 (2008).

7. Minch, K. J. *et al.* The DNA-binding network of Mycobacterium tuberculosis. *Nature Communications* **6**, 5829 (2015).

8. Pym, A. S. *et al.* Regulation of catalase-peroxidase (KatG) expression, isoniazid sensitivity and virulence by furA of Mycobacterium tuberculosis. *Molecular Microbiology* **40**, 879–889 (2001).

9. Zahrt, T. C., Song, J., Siple, J. & Deretic, V. Mycobacterial fura is a negative regulator of catalase–peroxidase gene katg. *Molecular Microbiology* **39**, 1174–1185 (2001).

10. Dudek, C.-A. & Jahn, D. PRODORIC: state-of-the-art database of prokaryotic gene regulation. *Nucleic Acids Research* **50**, D295–D302 (2021).

11. Hanamura, A. & Aiba, H. A new aspect of transcriptional control of the Escherichia coli crp gene: positive autoregulation. *Molecular Microbiology* **6**, 2489–2497 (2006).

12. Hanamura, A. & Aiba, H. Molecular mechanism of negative autoregulation of *Escherichia coli crp* gene. *Nucleic Acids Research* **19**, 4413–4419 (1991).

13. Fang, X. *et al.* Global transcriptional regulatory network for Escherichia coli robustly connects gene expression to transcription factor activities. *Proceedings of the National Academy of Sciences* **114**, 10286–10291 (2017).

14. Carrera, J. *et al.* An integrative, multi-scale, genome-wide model reveals the phenotypic landscape of Escherichia coli. *Molecular Systems Biology* **10**, 735–735 (2014).

15. Bennett, M. R. & Hasty, J. Microfluidic devices for measuring gene network dynamics in single cells. *Nature Reviews Genetics* **10**, 628–638 (2009).

16. Prindle, A. *et al.* A sensing array of radically coupled genetic 'biopixels'. *Nature* **481**, 39–44 (2012).

17. Rullan, M., Benzinger, D., Schmidt, G. W., Milias-Argeitis, A. & Khammash, M. An Optogenetic Platform for Real-Time, Single-Cell Interrogation of Stochastic Transcriptional Regulation. *Molecular Cell* **70**, 745–756.e6 (2018).

18. Wong, B. G., Mancuso, C. P., Kiriakov, S., Bashor, C. J. & Khalil, A. S. Precise, automated control of conditions for high-throughput growth of yeast and bacteria with eVOLVER. *Nature Biotechnology* **36**, 614–623 (2018).

19. Steel, H., Habgood, R., Kelly, C. L. & Papachristodoulou, A. In situ characterisation and manipulation of biological systems with Chi.Bio. *PLOS Biology* **18**, e3000794 (2020).

20. Bremer, H. & Dennis, P. P. Modulation of Chemical Composition and Other Parameters of the Cell at Different Exponential Growth Rates. *EcoSal Plus* **3**, ecosal.5.2.3 (2008).
