# Supplementary material for: Competition and evolutionary selection among core regulatory motifs in gene expression control
Source: Nat Commun. 2023 Dec 13;14:8266. doi: 10.1038/s41467-023-43327-7 (PMC10719253; doi:10.1038/s41467-023-43327-7)
Supplement: Supplementary file 2 — Reporting Summary [file 41467_2023_43327_MOESM2_ESM.pdf]

nature portfolio

Corresponding author(s): Andras Gyorgy
Last updated by author(s): Oct 13, 2023

Reporting Summary

Nature Portfolio wishes to improve the reproducibility of the work that we publish. This form provides structure for consistency and transparency in reporting. For further information on Nature Portfolio policies, see our Editorial Policies and the Editorial Policy Checklist.

Statistics

For all statistical analyses, confirm that the following items are present in the figure legend, table legend, main text, or Methods section.

n/a	Confirmed	
☐	☒	The exact sample size (n) for each experimental group/condition, given as a discrete number and unit of measurement
☒	☐	A statement on whether measurements were taken from distinct samples or whether the same sample was measured repeatedly
☐	☒	The statistical test(s) used AND whether they are one- or two-sided<br>*Only common tests should be described solely by name; describe more complex techniques in the Methods section.*
☒	☐	A description of all covariates tested
☒	☐	A description of any assumptions or corrections, such as tests of normality and adjustment for multiple comparisons
☐	☒	A full description of the statistical parameters including central tendency (e.g. means) or other basic estimates (e.g. regression coefficient) AND variation (e.g. standard deviation) or associated estimates of uncertainty (e.g. confidence intervals)
☐	☒	For null hypothesis testing, the test statistic (e.g. F, t, r) with confidence intervals, effect sizes, degrees of freedom and P value noted<br>*Give P values as exact values whenever suitable.*
☒	☐	For Bayesian analysis, information on the choice of priors and Markov chain Monte Carlo settings
☒	☐	For hierarchical and complex designs, identification of the appropriate level for tests and full reporting of outcomes
☒	☐	Estimates of effect sizes (e.g. Cohen's d, Pearson's r), indicating how they were calculated

Our web collection on statistics for biologists contains articles on many of the points above.

Software and code

Policy information about availability of computer code

Data collection	The manuscript does not rely on custom mathematical algorithms or software. Simulation data were generated and analyzed as described in the Methods using built-in MATLAB (version R2023a) functions. The MATLAB scripts used to obtain the results featured in the paper are publicly available at https://github.com/qbionet/evolutionary-selection. Additional information is available from the corresponding author upon request.
Data analysis	No custom software or code was utilized to analyze the data in this study. Simulation data were analyzed using built-in MATLAB (version R2023a) functions as described in the Methods. Figures were created using MATLAB (version R2023a) and Adobe Illustrator (version 26.5). Overleaf (version v2) online LaTeX editor was used to prepare and compile the manuscript.

For manuscripts utilizing custom algorithms or software that are central to the research but not yet described in published literature, software must be made available to editors and reviewers. We strongly encourage code deposition in a community repository (e.g. GitHub). See the Nature Portfolio guidelines for submitting code & software for further information.

Data

Policy information about availability of data

All manuscripts must include a data availability statement. This statement should provide the following information, where applicable:

- Accession codes, unique identifiers, or web links for publicly available datasets
- A description of any restrictions on data availability
- For clinical datasets or third party data, please ensure that the statement adheres to our policy

CoryneRegNet 7.0 and PRODORIC databases were downloaded from https://exbio.wzw.tum.de/coryneregnet/download.htm and https://www.prodoric.de/api/prodonet/, respectively. Data on the functional organization of the transcription regulatory network of E. coli were downloaded from https://www.pnas.org/doi/full/10.1073/pnas.1702581114. Source Data are provided with this paper.

Research involving human participants, their data, or biological material

Policy information about studies with human participants or human data. See also policy information about sex, gender (identity/presentation), and sexual orientation and race, ethnicity and racism.

Reporting on sex and gender	N/A
Reporting on race, ethnicity, or other socially relevant groupings	N/A
Population characteristics	N/A
Recruitment	N/A
Ethics oversight	N/A

Note that full information on the approval of the study protocol must also be provided in the manuscript.

Field-specific reporting

Please select the one below that is the best fit for your research. If you are not sure, read the appropriate sections before making your selection.

☒ Life sciences ☐ Behavioural & social sciences ☐ Ecological, evolutionary & environmental sciences

For a reference copy of the document with all sections, see nature.com/documents/nr-reporting-summary-flat.pdf

Life sciences study design

All studies must disclose on these points even when the disclosure is negative.

Sample size	Statistical methods were not used to predetermine sample sizes. For data pertaining to large populations, a single sample was considered due to the deterministic nature of the underlying mathematical model. For data corresponding to small populations, data points in each dataset range from 10 to 1000 to ensure that performance is evaluated when considering a diverse array of initial conditions and stochastic samples. In each case further increasing the sample size yielded no appreciable change in the results.
Data exclusions	No data were excluded from the analysis.
Replication	Replication is not applicable for this study as no experimental data were generated. For data pertaining to large populations, repetitions yielded identical results due to the deterministic nature of the underlying mathematical model. For data corresponding to small populations, replicating the computations produced nearly identical results except for minor variations (which are due to the stochastic sampling method) that do not impact the conclusions.
Randomization	Randomization is not applicable for this study as no experimental data were generated.
Blinding	Blinding is not applicable for this study as no experimental data were generated.

Reporting for specific materials, systems and methods

We require information from authors about some types of materials, experimental systems and methods used in many studies. Here, indicate whether each material, system or method listed is relevant to your study. If you are not sure if a list item applies to your research, read the appropriate section before selecting a response.

Materials & experimental systems

n/a	Involved in the study
☒	☐ Antibodies
☒	☐ Eukaryotic cell lines
☒	☐ Palaeontology and archaeology
☒	☐ Animals and other organisms
☒	☐ Clinical data
☒	☐ Dual use research of concern
☒	☐ Plants

Methods

n/a	Involved in the study
☒	☐ ChIP-seq
☒	☐ Flow cytometry
☒	☐ MRI-based neuroimaging
